# Impact of the kinetics of circulating anti-CD19 CAR-T cells and their populations on the outcome of DLBCL patients

**DOI:** 10.1038/s41408-024-01065-z

**Published:** 2024-05-17

**Authors:** Lourdes Martín-Martín, Sara Gutiérrez-Herrero, María Herrero-García, Alejandro Martín García-Sancho, Ana Yeguas, Ana-África Martín-López, Lucía López-Corral, Estefanía Pérez-López, Marta García-Blázquez, Fermín Sánchez-Guijo, María Belén Vidriales, Giuseppe Gaipa, INCAR consortium, Alberto Orfao, EuroFlow consortium, María Belén Vidriales, Alberto Orfao

**Affiliations:** 1 Translational and Clinical Research Program, Centro de Investigación del Cáncer and Instituto de Biología Molecular y Celular del Cáncer (IBMCC), Consejo Superior de Investigaciones Científicas (CSIC), and University of Salamanca, Salamanca, Spain; 2 Flow Cytometry Service (NUCLEUS), University of Salamanca, Salamanca, Spain; 3 Institute of Biomedical Research of Salamanca (IBSAL), Salamanca, Spain; 4 Department of Medicine, University of Salamanca (Universidad de Salamanca), Salamanca, Spain; 5 Biomedical Research Networking Centre Consortium of Oncology (CIBERONC), Carlos III Health Institute, Madrid, Spain; 6 Department of Hematology, University Hospital of Salamanca, Salamanca, Spain; 7 Tettamanti Center and Pediatrics, Fondazione IRCCS San Gerardo dei Tintori, Monza, Italy

**Keywords:** B-cell lymphoma, Cancer immunotherapy

Dear Editor,

CAR-T cell therapy has led to a significant advance in the treatment of refractory/relapsed diffuse large B-cell lymphoma (DLBCL) [1]. However, only less than half of all CAR-T-treated DLBCL patients achieve long-term disease control [2–4]. Among other parameters, the efficacy of CAR-T therapy in DLBCL has been associated with the patients’ immune system, the composition of the CAR-T product [5], the magnitude of the in vivo expansion and persistence of CAR-T cells [6]. Here, we used next-generation flow cytometry to investigate the kinetics of circulating anti-CD19 CAR-T cells and their populations in blood, and to determine their potential utility for predicting response to therapy. For this purpose, we studied 58 relapsed/refractory DLBCL patients (36 men and 22 women; median [range] age, 62 [32–79] years) treated with anti-CD19 axicabtagene ciloleucel (axi-cel, Kite, Gilead, Santa Monica, CA) or tisagenlecleucel (tisa-cel, Novartis, Bâle, Switzerland) CAR-T cells (Tables S1, S2; Supplementary Data 1).

Globally, anti-CD19 CAR-T cells peaked at day +7 (50%) or day +14 (43%) post-infusion with a median (range) number at the peak of 68 (0.5–1771) CAR-T cells/µL and consisted of >75 distinct populations, from which 12/75 CAR-T cell subsets were highly prevalent (Figure S1A). CAR-TCD4^+^ cells predominated (median: 40 cells/µL) over CAR-TCD8^+^ cells (26 cells/µL), with a balanced (median) distribution of 45% vs. 54%, respectively (Table S3). Overall, central memory (CM) T-cells predominated both among CAR-TCD4^+^ (17 cells/µL) and CAR-TCD8^+^ cells (18 cells/µL), followed by CAR-TCD8^+^ transitional memory (TM) (4.3 cells/µL) and CAR-TCD4^+^ effector memory (EM) cells (3.8 cells/ µL). In turn, CAR-T helper 1 (Th1) cells (11 cells/µL), CAR-Th1/Th2 (4.9 cells/µL), and regulatory (2.2 cells/µL) CAR-T cells (Tregs) were the most expanded functional CAR-TCD4^+^ populations; other CAR-T cell populations typically comprised (median values of) <1 cell/µL (Figure S1B; Table S3). Interestingly, significantly different numbers of CAR-T cell subsets were also observed at their peak, depending on the commercial product used (Tables S4, S5). Thus, axi-cel CAR-T cells (*n* = 33) tended to reach their maximum expansion (*C*_max_) earlier than tisa-cel (*n* = 25) CAR-T cells (day+7 vs. day +14, respectively) (Table S6; Figure S2A), with significantly higher median CAR-T cell counts (113 vs. 34 cells/µL for axi-cel vs. tisa-cel; *p* = 0.01) (Table S6; Figure S2B) and a significantly different composition, but similar median CAR expression levels/cell (Tables S4–S6; Figure S2C-D).

As expected, an inverse correlation was observed along the whole monitoring period between the number of CAR-T cells in blood and that of both circulating lymphoma cells (CLC) (*r* = −0.2; *p* < 0.001) and normal residual B-lymphocytes (*r* = −0.3; *p* < 0.001), a sustained recovery from B-cell aplasia after the CAR-T decline being observed in only 8/58 (14%) patients (Figure S3A–D). In another 10/58 patients (17%), transient emergence of <5 normal residual B-lymphocytes/µL (median: 0.4 vs. 72 B-cells/µL for patients who lost B-cell aplasia; *p* < 0.001), followed by a recovery/increase in circulating CAR-T cell counts, in the absence of a maintained recovery of normal B-lymphocytes, was detected (Figure S3E, F).

Each patient was followed for ≥3 months post-infusion, except for those 7 cases who failed to respond to CAR-T cell therapy and died prior to that time point with an overall response (OR) rate of 67% (95% CI, 54–79%): 33/58 (57%, 95% CI, 43–70%) patients achieved complete response (CR) after a median of 30 days and 6/58 (10%) showed partial response (PR), whereas 19/58 (33%) patients did not respond (NR) and had progressive disease (Figure S4). The median duration of response was not reached and a sustained CR of up to 4.5 years was documented in 30 patients after a median follow-up of 2 years post-infusion. At the study closure, the majority of DLBCL patients (30/58, 52%) showed durable CR, 25 (43%) did not reach CR, and 3 (5.2%) had CR followed by disease recurrence after 4, 8 and 10 months, respectively. Consistent with previous reports [7, 8], CR patients more frequently achieved the CAR-T peak at an earlier time point (day +7: 55% vs. 48%, respectively) (Fig. 1A) and showed a greater expansion of CAR-T cells vs. PR/NR cases (132 vs. 29 cells/µL, respectively; *p* = 0.002) (Fig. 1B, C; Table S7). This translated into a significantly prolonged median CAR-T cell lifespan in blood in CR vs. PR/NR patients (8.3 vs. 1 months, respectively; *p* < 0.001) (Fig. 1D) with detectable CAR-T cells in blood even for >3 years post-infusion in the former group (Fig. 1A). Importantly, patients who maintained detectable CAR-T cells (≥0.01 cells/µL) after achieving CR, also showed significantly higher rates of sustained CR (Fig. 2A). These findings translated into a higher median expansion of CAR-T cells between days +1 to +28 post-infusion in CR vs. PR/NR cases, calculated as the area under the curve (AUC_0–28_): 1505 vs. 270 days x cells/µL, respectively (*p* = 0.001). Likewise, CR patients also showed greater numbers in blood of most CAR-T cell populations vs. PR/NR DLBCL, including higher median numbers (cells/µL) of CAR-TCD4^+^ (46 vs. 15; *p* = 0.006), CAR-TCD8^+^ (53 vs. 9.2; *p* = 0.001) and CAR-Tγδ^+^ (0.2 vs. <0.001; *p* < 0.001)%–and their maturation-associated CAR-TCD4^+^_CM_ (25 vs. 5.3; *p* = 0.002), CAR-TCD8^+^_CM_ (33 vs. 4.4; *p* = 0.002) and CAR-Tγδ^+^_CM_ (0.2 vs. <0.001; *p* < 0.001) subsets-, in addition to greater CAR-TFH (0.2 vs. <0.001; *p* < 0.03), CAR-Th1 (22 vs. 4.3; *p* < 0.001) and CAR-Th1/Th2 (9.9 vs. 1.8; *p* = 0.02) functional subset counts, among other minor CAR-T cell populations (Fig. 1C; Tables S7, S8).

**Fig. 1:**
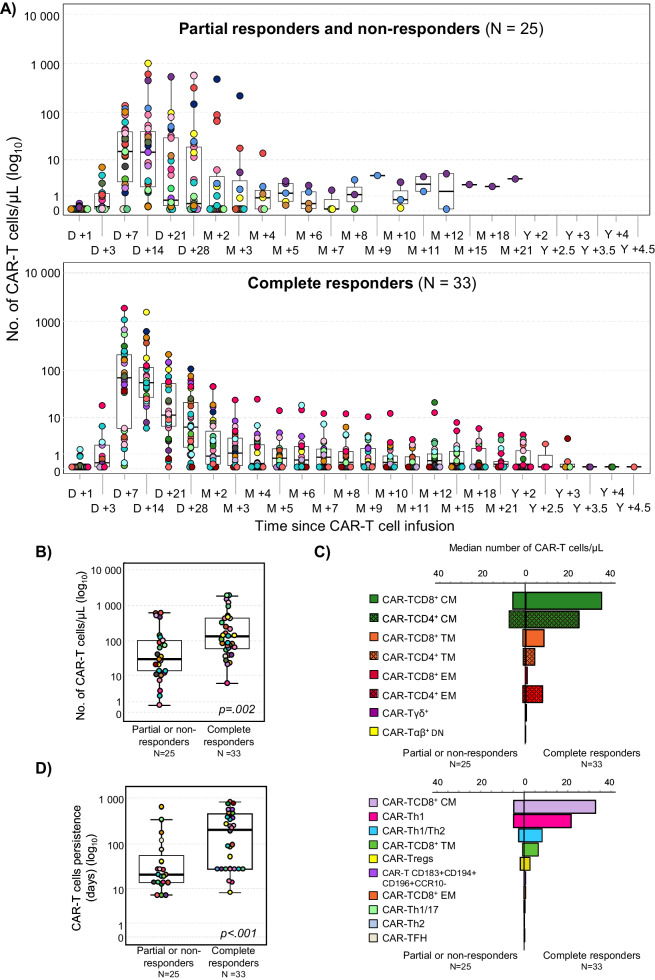
Differences in CAR-T-cell kinetics and composition at the CAR-T peak in blood of partial responder plus non-responder vs. complete responder DLBCL patients and its impact on progression-free survival. **A** Comparison between the kinetics of circulating anti-CD19 CAR-T cells in blood of (color-coded) individual patients grouped according to response to therapy (partial responders plus non-responders vs. complete responders) studied at predefined time points during follow-up. Number (**B**), composition (**C**) and persistence (**D**) of anti-CD19 CAR-T cells circulating in blood of DLBCL patients grouped according to response to CAR-T therapy (partial responders plus non-responders vs. complete responders). D day, M month, Y year, CM central memory, TM transitional memory, EM effector memory, TE terminal effector, TFH T follicular helper cells, Th T helper cells, Tregs T regulatory cells.

**Fig. 2:**
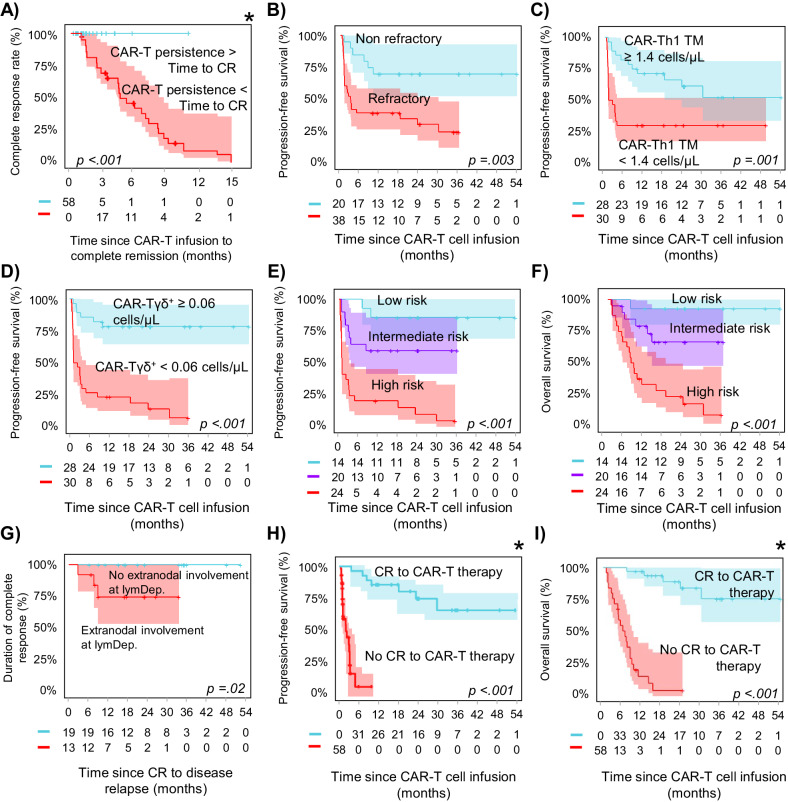
Clinical, biological and CAR-T cell features with an independent prognostic value on disease response to therapy, progression-free survival and overall survival of DLBCL patients after CAR-T cell therapy. **A** Impact of CAR-T cell persistence on the achievement of complete response (CR); **B**–**D** progression-free survival probability according to those variables with an independent impact on the achievement of complete response; **E** prognostic impact of the here proposed scoring system for predicting CR on DLCBL patient progression-free survival and **F** overall survival; **G** prognostic impact of extranodal involvement at lymphodepletion on disease recurrence; prognostic impact of CR on **H** progression-free survival and **I** overall survival. LymDep lymphodepletion. *Simon–Makuch plot.

Interestingly, 8/8 patients who experienced B-cell recovery after CAR-T therapy achieved CR (vs. 0/25 PR/NR cases; *p* = 0.007), one of them showing disease relapse 8 months post-infusion (Figure S5). Conversely, the presence of CLC at apheresis, or right before infusion, did not affect treatment response (Figure S4). Noteworthy, axi-cel-treated patients showed higher CR rates (*p* = 0.02 vs patients infused with tisa-cel), although, significant differences were restricted to older (≥60 years) patients (*p* = 0.004) (Table S6; Figures S4 and S6).

Multivariate analysis identified the combination of the disease status prior to apheresis (OR: 11.3; 95% CI: 1.7–74), and the number of CAR-Tγδ^+^ cells/µL (OR: 8.8; 95% CI: 1.9–41) and CAR-Th1_TM_ cells/µL (OR: 6.2; 95% CI: 1.4–29) at the peak, as independent predictors for CR (Fig. 2B–D and S4). Based on these three variables, a scoring system was constructed to predict for CR in which the presence of refractory disease at apheresis scored 2 points, lower CAR-Tγδ+ counts (<0.06 cells/µL) at the peak scored 1.5, and CAR-Th1_TM_ numbers <1.4 cells/µL scored 1 point (vs. 0 for the other cases). Subsequently, DLBCL patients were stratified at the CAR-T cell peak into low-risk (score: 0–1, 14 patients [24%]), intermediate (score 1.5–3, 20 patients [35%]) and high-risk (score ≥3.5, 24 patients [41%]) DLBCL, with decreasing CR rates of 100%, 70% (OR: 0.5; 95% CI: 0.4–0.7) and only 21% (OR: 0.3; 95% CI: 0.1–0.6), respectively (*p* < 0.001) (Fig. 2E–F).

At the study closure, median PFS was 10 months with a 2-year PFS rate of 45% (95% CI: 33–60%). Most patients who achieved CR showed sustained response (30/33, 91%), resulting in 2-year and 4-year CR rates of 90% (95% CI: 81–100%). One patient relapsed with CD19-negative cells, another with CD19-positive cells, and in the third case, a relapse was detected by PET scan, but could not be confirmed histologically. Extranodal involvement at lymphodepletion emerged as the sole factor impacting disease recurrence among CR patients, since all three CR patients who experienced a relapse had extranodal involvement at lymphodepletion, whereas none of those without it relapsed (0/19) (*p* = 0.03) (Fig. 2G). At study closure, 27/58 (47%) patients had died due to disease progression (23/27, 85%) and infection (4/27, 15%), with a median OS of 2.5 years and a 2-year OS rate of 52% (95% CI: 40–66%). As expected, those parameters associated with CR, also emerged as prognostic features for both PFS and OS (Fig. 2E–F and S6, S7).

CR was the sole independent predictor for PFS and OS (*p* < 0.001 for both) (Fig. 2H–I and S6, S7), with significant differences in the kinetics and composition of CAR-T cells at their peak in blood of CR patients vs. the other cases. Importantly, the number of CAR-Tγδ^+^ and CAR-Th1_TM_ cells, together with the disease status at apheresis, emerged early (at the CAR-TCD19 peak) as independent predictors for the identification of DLBCL patients at high risk of treatment failure (vs. CR). These results highlight the relevance of CAR-T cell monitoring in the management of CAR-T-treated DLBCL for guiding early therapeutic decisions, and point out for the first time, a critical role of CAR-Tγδ^+^ cells—currently depleted from the leukapheresis-derived CAR-T cell products [9]—in response to therapy. The clinical relevance of TCRγδ^+^ cells in CAR-T cell-treated DLBCL patients might be not only due to their potent cytotoxic ability and proven antitumor activity [10], even in the absence of HLA-mediated antigen presentation [11, 12], but also to their ability to migrate from blood to peripheral tissues where extranodal involvement by lymphoma cells frequently occurs in relapsed/refractory DLBCL [13, 14]. Additionally, unlike standard anti-CD19 CAR-Tαβ^+^ cells, CAR-Tγδ^+^ cells have demonstrated reactivity in vitro and in vivo against both CD19-positive and CD19-negative tumor cells [15], suggesting that CAR-Tγδ^+^ cells might target CD19 tumor cells even after antigen loss, and retain specificity via their TCR.

Our findings highlight the complexity and diversity of T-cell responses following CAR-T therapy, which may not solely depend on CAR-Tαβ^+^ cells. Thus, selection or depletion of specific CAR-T cell populations (e.g., CAR-Tγδ^+^ cells) during the manufacturing process, and thereby also from the CAR-T cell product, might contribute to an increase treatment success vs. failure, which emphasizes the value of the ex vivo characterization and monitoring of CAR-T cells in blood together with the disease status at apheresis, for early identification of DLBCL patients at risk of treatment failure.

### Supplementary information

Supplemental material

## Data Availability

Data generated or analyzed during this study are included in this published article (and its supplementary information files). Any other data will be provided on reasonable request to the corresponding author.
