# Supplementary material for: Impact of the kinetics of circulating anti-CD19 CAR-T cells and their populations on the outcome of DLBCL patients
Source: Blood Cancer J. 2024 May 17;14(1):83. doi: 10.1038/s41408-024-01065-z (PMC11101460; doi:10.1038/s41408-024-01065-z)
Supplement: Supplementary file 1 — Supplemental material [file 41408_2024_1065_MOESM1_ESM.pdf]

Supplementary data 1.

SUBJECTS, MATERIALS AND METHODS

Patients. A total of 58 refractory/relapsed DLBCL adult (≥18y) patients who were treated with anti-CD19 CAR-T cells at the University Hospital of Salamanca (Spain) between May 2019 and January 2023 were studied. All patients had an histologically confirmed diagnosis of relapsed/refractory DLBCL according to the World Health Organization (WHO) 2016 classification (1) after ≥2 lines of systemic treatment, except for 4 patients included in the ZUMA-7 (N=2) and BELINDA (N=2) clinical trials (2, 3), (ClinicalTrials.gov #NCT03391466 and #NCT03570892) who had only received one line of treatment prior to CAR-T cells. All patients were judged eligible for commercial anti-CD19 CAR-T cell therapy by the Expert Committee of the Spanish National Health System, except for the four patients enrolled in the ZUMA-7 or BELINDA trials. Prior to enrollment, each patient gave his/her written informed consent to participate in the study and the study was approved by the local Ethics Committee (code: PI 2019 05 314).

CAR-T cell therapy consisted of either axicabtagene ciloleucel (axi-cel, Kite, Gilead, Santa Monica, CA) or tisagenlecleucel (tisa-cel, Novartis, Bâle, Switzerland). Prior to leukapheresis and CAR-T cell infusion, patients were monitored for the presence of circulating lymphoma cells (CLC) in blood. After CAR-T infusion, blood monitoring for CAR-T cells and their subpopulations was additionally performed, weekly during the first month post-infusion, monthly during the first year, every 3 months during the second year, and at 6-month intervals, thereafter. PET-CT for evaluation of response was performed after bridging therapy (before lymphodepletion) and at months 1, 3, 6, 12, 18 and 24 after anti-CD19 CAR-T infusion.

End points and clinical assessment. Primary endpoints were overall and complete response rates (determined following the 2014 Lugano response criteria (4) for patients who had ≥3 months follow-up or those who failed to respond to CAR-T cell therapy and died prior to that time point). Progression-free survival (PFS), duration of complete

response (DOCR; time from CR to the date of recurrence) and overall survival (OS) were secondary endpoints.

Immunophenotypic studies. Prospective monitoring of CLC, CAR-T cells and other immune cells was sequentially performed in blood by next generation flow cytometry (NGF) for each DLBCL patient (N=58), for a total of 840 samples (median: 14 samples/patient; range: 6-25). For this purpose, 10 mL of fresh (<12h) blood was collected in K3-EDTA Vacutainer tubes -Becton/Dickinson Biosciences (BD), New Jersey, NJ- per time point per patient, aliquoted, and stained with the EuroFlow Lymphocyte Screening Tube (LST) (5), and both the immune monitoring (IMM) T $CD4^{+}$ (TCD4) and T/NK-cell cytotoxic (Cytox) tubes, to which the CD19 protein (20-291)-FITC reagent from ACROBiosystems (Bethesda, MD) was added. Sample staining was performed according to the EuroFlow standard procedures available at www.euroflow.org, for staining of cell surface membrane-only and cell surface membrane plus cytoplasmic markers, as previously described (6, 7). These antibody combinations allowed the identification of ≥85 subsets of $CD4^{+}$ T-cells and 48 subsets of $CD8^{+}$ T, $TCR\gamma\delta^{+}$ and NK cells, within both CAR-T cells and normal residual T/NK lymphocytes. Stained samples were measured on either a FACSCanto II (BD) flow cytometer (for the LST tube) or a 3-laser Aurora (Cytek, Fremont, CA) flow cytometer (for the TCD4 and Cytox IMM tubes) using the FACSDiva (BD) and the SpectroFlo (Cytek) software programs, respectively. For data analysis the Infinicyt software (Cytognos, Salamanca, Spain) was used (5-7).

Statistical methods. Two-group comparisons for continuous variables were made using either the two-tailed Mann-Whitney U test or the Wilcoxon rank sum test for unpaired or paired data, respectively; for categorical variables, the $\chi 2$ test was used. The association between continuous variables was assessed by the Spearman correlation. Receiver operating characteristic (ROC) curves were built using the empirical method, and optimal cut-off values established based on the Youden index. Univariate odds ratio (OR) and a

multivariate logistic regression model were applied to identify the different covariates associated with response to therapy. For time-fixed covariates, progression-free survival (PFS) and overall survival (OS) curves were plotted according to the Kaplan-Meier method and compared using the (one-sided) log-rank test. In turn, PFS curves for time-varying covariates were plotted using the Simon-Makuch method and contrasted with the Mantel-Byar test (R statistical software v4.3.0) (8). CAR-T cell persistence and time to B-cell recovery were evaluated using the cumulative incidence method, considering relapse or death as competing risks. CAR-T cell lifespan was evaluated by censoring the subjects with detectable CAR-T cells who had discontinued participation in the study due to CAR-T cell response failure or death. The variables that demonstrated a significant impact on survival in the univariate analyses were further submitted to a multivariate analysis using a Cox stepwise regression model (Schönfeld residuals were used to check the proportional hazards assumption). Time of CAR-T cell infusion was used as the origin in all time-to-event analyses. For all statistical analyses the Statistical Package for Social Sciences (SPSS, v26; BM, Armonk, NY) was used, except if otherwise specified. Statistical significance was set at *p* values ≤0.05.

SUPPLEMENTARY REFERENCES:

1. Swerdlow SH, Campo E, Pileri SA, Harris NL, Stein H, Siebert R, et al. The 2016 revision of the World Health Organization classification of lymphoid neoplasms. Blood. 2016;127(20):2375-90.
2. Locke FL, Miklos DB, Jacobson CA, Perales MA, Kersten MJ, Oluwole OO, et al. Axicabtagene Ciloleucel as Second-Line Therapy for Large B-Cell Lymphoma. N Engl J Med. 2022;386(7):640-54.
3. Bishop MR, Dickinson M, Purtill D, Barba P, Santoro A, Hamad N, et al. Second-Line Tisagenlecleucel or Standard Care in Aggressive B-Cell Lymphoma. N Engl J Med. 2022;386(7):629-39.
4. Cheson BD, Fisher RI, Barrington SF, Cavalli F, Schwartz LH, Zucca E, et al. Recommendations for initial evaluation, staging, and response assessment of Hodgkin and non-Hodgkin lymphoma: the Lugano classification. J Clin Oncol. 2014;32(27):3059-68.
5. Flores-Montero J, Grigore G, Fluxá R, Hernández J, Fernandez P, Almeida J, et al. EuroFlow Lymphoid Screening Tube (LST) data base for automated identification of blood lymphocyte subsets. J Immunol Methods. 2019;475:112662.
6. Kalina T, Flores-Montero J, van der Velden VH, Martin-Ayuso M, Böttcher S, Ritgen M, et al. EuroFlow standardization of flow cytometer instrument settings and immunophenotyping protocols. Leukemia. 2012;26(9):1986-2010.

7. Flores-Montero J, Sanoja-Flores L, Paiva B, Puig N, García-Sánchez O, Böttcher S, et al. Next Generation Flow for highly sensitive and standardized detection of minimal residual disease in multiple myeloma. Leukemia. 2017;31(10):2094-103.
8. Simon R, Makuch RW. A non-parametric graphical representation of the relationship between survival and the occurrence of an event: application to responder versus non-responder bias. Stat Med. 1984;3(1):35-44.

Supplementary Table S1. Baseline clinical and laboratory characteristics of DLBCL patients included in this study (N=58).

Baseline features	Patient features (N=58)
**Age,** years	62 (32 - 79)
**Gender**	
Male	36 (62%)
Female	22 (38%)
**2016 WHO classification**	
Diffuse large B-cell lymphoma (DLBCL, NOS)	47 (81%)
High grade B-cell lymphoma (HGBL) (*MYC* and *BCL2* and/or *BCL6* translocations)	5 (9%)
Transformed follicular lymphoma	6 (10%)
**Molecular subtypes** (based on the Hans algorithm)	
Germinal center	27 (51%)
Non-germinal center (e.g. activated B-cell type)	26 (47%)
**Pre-apheresis**	
**Previous therapy**	2 (1 - 7)
< 3 lines	47 (81%)
≥ 3 lines	11 (19%)
**Previous aHSCT**	
No	47 (81%)
Yes	11 (19%)
**Disease status**	
Non-refractory to last line of therapy	20 (34%)
Refractory to last line of therapy	38 (66%)
**Circulating lymphoma cells (CLC)**	
No	48 (83%)
Yes	8 (14%)
**Bulky mass** ≥ 7 cm	
No	45 (78%)
Yes	13 (22%)
**IPI**	
0-1 Low risk	15 (26%)
2-3 Intermediate risk	38 (65%)
4-5 High risk	5 (9%)
**ECOG performance status**	1 (0 - 2)
0	17 (29%)
1	39 (68%)
2	2 (3%)
**Disease stage**	
I / II	18 (31%)
III / IV	40 (69%)
**LDH*,** IU/L	258 (127 - 1934)
Normal (< 280)	31 (53%)
Elevated (≥ 280)	27 (47%)
**Ferritin***, ng/mL	512 (55 - 2165)
Normal (< 300 in male and < 200 in female)	13 (22%)
Elevated (≥ 300 in male and ≥ 200 in female)	45 (78%)
**Detectable B cells in blood**	
No	43 (74%)
Yes	14 (24%)
**Pre-lymphodepletion**	
**Bulky mass** ≥ 7 cm	
No	45 (78%)
Yes	13 (22%)
**IPI**	
0-1 Low risk	17 (31%)
2-3 Intermediate risk	30 (54%)
4-5 High risk	8 (15%)
**MTV *,** cm^3	99 (0 - 2539)
**TLG *,** SUV x cm^3	827 (0 - 27213)
**Bone marrow involvement**	
No	40 (69%)
Yes	18 (31%)
**CNS involvement**	
No	56 (97%)
Yes	2 (3%)
**Extranodal involvement**	
No	25 (43%)
Yes	32 (55%)
**Disease stage**	
I / II	15 (26%)
III / IV	40 (69%)
**LDH*,** IU/L	242 (132 - 1013)
Normal (< 280)	33 (57%)
Elevated (≥ 280)	25 (43%)
**Ferritin***, ng/mL	462 (15 - 4432)
Normal (< 300 in male and < 200 in female)	16 (28%)
Elevated (≥ 300 in male and ≥ 200 in female)	42 (72%)
**Bridging therapy**	
No	11 (19%)
Yes	47 (81%)
**Pre-Infusion**	
**Ferritin*,** ng/mL	688 (14 - 5217)
Normal (< 300 in male and < 200 in female)	10 (17%)
Elevated (≥ 300 in male and ≥ 200 in female)	48 (83%)
**Circulating lymphoma cells (CLC)**	
No	52 (90%)
Yes	6 (10%)
**Detectable B cells in blood**	
No	52 (90%)
Yes	6 (10%)

Results expressed as number and percentage of patients or *as median number and range. aHSCT, autologous hematopoietic stem cell transplant; CNS, central nervous system; ECOG, Eastern Cooperative Oncology Group; IPI, international prognostic index; LDH, lactate; dehydrogenase; MTV, metabolic tumor volume; TLG, total lesion glycolysis.

Supplementary Table S2. Overview of those therapies used prior to anti-CD19 CAR-T cell infusion and of the bridging therapies administered to the DLBCL patients included in this study (N=58).

Antitumor therapy	Previous lines (N=58)								Bridging therapy (N=47)
	1st line	2nd line	3rd line	4th line	5th line	6th line	7th line	Other	
CHOP-based protocol (CHOP/CCOP/COMP/CVP) ± R	44 (76%)	2 (3.4%)			1 (1.7%)	1 (1.7%)			2 (4.8%)
Platinum-based protocol (ESHAP/DHAP/GDP) ± R	2 (3.4%)	32 (55%)	5 (8.6%)						
GEMOX ± R	2 (3.4%)	9 (16%)	2 (3.4%)	1 (1.7%)					19 (45%)
R-EPOCH	8 (14%)	1 (1.7%)							
Ifosfamide-based protocol (ICE/IFE/IE) ± R		8 (13.8%)	2 (3.4%)						
BURKIMAB-13 protocol	2 (3.4%)								
R-Lenalidomide		1 (1.7%)							2 (4.8%)
R-IE + Ibrutinib		1 (1.7%)							
Pembrolizumab			1 (1.7%)						
Polatuzumab + Bendamustine ± R			1 (1.7%)						7 (17%)
Bendamustine-based protocol ± R				1 (1.7%)	1 (1.7%)	1 (1.7%)			4 (9.5%)
BV-Nivolumab							1 (1.7%)		1 (2.4%)
Other antineoplastic drugs			2 (3.4%)	3 (5.2%)	1 (1.7%)	1 (1.7%)	1 (1.7%)		1 (2.1%)
Radiotherapy								7 (12%)	-
aHSCT								11 (19%)	-
**TOTAL**	**58 (100%)**	**54 (93%)**	**13 (22%)**	**5 (8.6%)**	**3 (5.2%)**	**3 (5.2%)**	**2 (3.4%)**	**18 (31%)**	**36 (62%)**
**Other therapies (corticosteroids)**		2 (3.4%)	1 (1.7%)						11 (26%)

aHSCT: autologous hematopoietic stem cell transplantation; BURKIMAB: rituximab, metotrexate, dexamethasone, ifosfamide, vincristine, etoposide, cytarabine, doxoribicin, vindesine; BV: brentuximab vedotin; CCOP: cytoxan, pegylated liposomal doxorubicin (PLD), vincristine, prednisone; CHOP: cyclophosphamide, doxorubicin, vincristine, prednisone; COMP: prednisone, cyclophosphamide, vincristine, myocet™; CVP: cyclophosphamide, vincristine, prednisone; DHAP: dexamethasone cytarabine cisplatin; EPOCH: etoposide, prednisone, vincristine, cyclophosphamide, doxorubicin; ESHAP: etoposide, cisplatin and cytarabine; GDP: gemcitabine, dexamethasone, cisplatin; GEMOX: gemcitabine oxaliplatin; ICE: ifosfamide, carboplatin and etoposide; IE: ifosfamide, etoposide; IFE: ifosfamide, etoposide, epirubicin; R, rituximab.

Supplementary Table S3. Distribution of anti-CD19 CAR-T cells and their subpopulations in blood of DLBCL patients (N=58) at the CAR-T peak.

	cells/µL	%
**CAR-T cells**	68 (0.5 - 1771)	20 (3 - 91)
**CAR-Tαβ$^+$ CD4$^+$**	40 (0.02 - 631)	45 (0.1 - 100)
**Maturation-associated subsets**		
CAR-Tαβ$^+$ CD4$^+$ CM	17 (<0.001 - 478)	23 (<0.001 - 82)
CAR-Tαβ$^+$ CD4$^+$ TM	2 (<0.001 - 117)	3 (<0.001 - 21)
CAR-Tαβ$^+$ CD4$^+$ EM	3.8 (<0.001 - 115)	3.3 (<0.001 - 44)
CAR-Tαβ$^+$ CD4$^+$ TE	<0.001 (<0.001 - 1.1)	<0.001 (<0.001 - 1.6)
**Functional (and maturation)-associated subsets**		
CAR-TFH	0.07 (<0.001 - 9.5)	0.07 (<0.001 - 2.8)
CAR-TFH Regulatory	<0.001 (<0.001 - 0.4)	<0.001 (<0.001 - 0.5)
CAR-TFH Th1 Like	<0.001 (<0.001 - 5.9)	<0.001 (<0.001 - 2.2)
CAR-TFH Th2 Like	<0.001 (<0.001 - 0.08)	<0.001 (<0.001 - 0.1)
CAR-TFH Th1/Th2 Like	<0.001 (<0.001 - 6.4)	<0.001 (<0.001 - 1.3)
CAR-TFH Th17 Like	<0.001 (<0.001 - 0.05)	<0.001 (<0.001 - 0.05)
CAR-TFH Th1/Th17 Like	<0.001 (<0.001 - 0.3)	<0.001 (<0.001 - 0.1)
CAR-TFH CD183+ CD194+ CD196+ CCR10-	<0.001 (<0.001 - 1)	<0.001 (<0.001 - 0.3)
CAR-Tregs	2.2 (<0.001 - 120)	1.7 (<0.001 - 68)
CAR-Tregs Th1 Like	0.5 (<0.001 - 69)	0.5 (<0.001 - 40)
CAR-Tregs Th2 Like	<0.001 (<0.001 - 0.8)	<0.001 (<0.001 - 0.7)
CAR-Tregs Th1/Th2 Like	0.8 (<0.001 - 48)	0.5 (<0.001 - 35)
CAR-Tregs Th17 Like	<0.001 (<0.001 - 0.4)	<0.001 (<0.001 - 0.3)
CAR-Tregs Th1/Th17 Like	<0.001 (<0.001 - 1.4)	<0.001 (<0.001 - 0.8)
CAR-Tregs CD183+ CD194+ CD196+ CCR10-	<0.001 (<0.001 - 3.9)	<0.001 (<0.001 - 1.6)
CAR-Th1	11 (<0.001 - 482)	20 (<0.001 - 84)
CAR-Th1 CM	6.8 (<0.001 - 363)	12 (<0.001 - 67)
CAR-Th1 TM	1.1 (<0.001 - 74)	1.6 (<0.001 - 16)
CAR-Th1 EM	1.4 (<0.001 - 115)	1.9 (<0.001 - 33)
CAR-Th1 TE	<0.001 (<0.001 - 1)	<0.001 (<0.001 - 1.5)
CAR-Th2	0.04 (<0.001 - 18)	0.01 (<0.001 - 18)
CAR-Th2 CM	0.03 (<0.001 - 14)	0.009 (<0.001 - 14)
CAR-Th2 TM	<0.001 (<0.001 - 1.1)	<0.001 (<0.001 - 1.1)
CAR-Th2 EM	<0.001 (<0.001 - 6.7)	<0.001 (<0.001 - 3.6)
CAR-Th2 TE	<0.001 (<0.001 - 0.03)	<0.001 (<0.001 - 0.04)
CAR-Th1/Th2	4.9 (<0.001 - 354)	7.1 (<0.001 - 64)
CAR-Th1/Th2 CM	3.8 (<0.001 - 276)	5.2 (<0.001 - 49)
CAR-Th1/Th2 TM	0.3 (<0.001 - 78)	0.3 (<0.001 - 14)
CAR-Th1/Th2 EM	0.3 (<0.001 - 18)	0.4 (<0.001 - 12)
CAR-Th1/Th2 TE	<0.001 (<0.001 - 0.05)	<0.001 (<0.001 - 0.1)
CAR-Th17	<0.001 (<0.001 - 10)	<0.001 (<0.001 - 10)
CAR-Th17 CM	<0.001 (<0.001 - 4.2)	<0.001 (<0.001 - 4.2)
CAR-Th17 TM	<0.001 (<0.001 - 0.5)	<0.001 (<0.001 - 0.7)
CAR-Th17 EM	<0.001 (<0.001 - 5.8)	<0.001 (<0.001 - 5.8)
CAR-Th1/Th17	0.2 (<0.001 - 21)	0.3 (<0.001 - 11)
CAR-Th1/Th17 CM	0.1 (<0.001 - 12)	0.1 (<0.001 - 2.4)
CAR-Th1/Th17 TM	<0.001 (<0.001 - 6.9)	<0.001 (<0.001 - 1.5)
CAR-Th1/Th17 EM	0.1 (<0.001 - 5.8)	0.1 (<0.001 - 8.1)
CAR-Th22	<0.001 (<0.001 - 0.2)	<0.001 (<0.001 - 0.2)
CAR-Th22 CM	<0.001 (<0.001 - 0.07)	<0.001 (<0.001 - 0.07)
CAR-Th22 EM	<0.001 (<0.001 - 0.1)	<0.001 (<0.001 - 0.1)
CAR-T CD183+ CD194+ CD196+ CCR10+	<0.001 (<0.001 - 0.5)	<0.001 (<0.001 - 4)
CAR-T CD183+ CD194+ CD196+ CCR10+ CM	<0.001 (<0.001 - 0.3)	<0.001 (<0.001 - 3)
CAR-T CD183+ CD194+ CD196+ CCR10+ TM	<0.001 (<0.001 - 0.09)	<0.001 (<0.001 - 0.2)
CAR-T CD183+ CD194+ CD196+ CCR10+ EM	<0.001 (<0.001 - 0.2)	<0.001 (<0.001 - 1)
CAR-T CD183+ CD194+ CD196+ CCR10-	0.6 (<0.001 - 28)	0.4 (<0.001 - 31)
CAR-T CD183+ CD194+ CD196+ CCR10- CM	0.2 (<0.001 - 16)	0.1 (<0.001 - 10)
CAR-T CD183+ CD194+ CD196+ CCR10- TM	0.4 (<0.001 - 131)	<0.001 (<0.001 - 5.3)
CAR-T CD183+ CD194+ CD196+ CCR10- EM	0.1 (<0.001 - 14)	<0.001 (<0.001 - 19)
CAR-T CD183+ CD194+ CD196+ CCR10- TE	<0.001 (<0.001 - 0.02)	<0.001 (<0.001 - 0.05)
CAR-T CD183+ CD194+ CD196- CCR10+	<0.001 (<0.001 - 0.8)	<0.001 (<0.001 - 1.5)
CAR-T CD183+ CD194+ CD196- CCR10+ CM	<0.001 (<0.001 - 0.7)	<0.001 (<0.001 - 0.8)
CAR-T CD183+ CD194+ CD196- CCR10+ TM	<0.001 (<0.001 - 0.1)	<0.001 (<0.001 - 0.4)
CAR-T CD183+ CD194+ CD196- CCR10+ EM	<0.001 (<0.001 - 0.08)	<0.001 (<0.001 - 0.2)
CAR-T CD183- CD194+ CD196- CCR10+	<0.001 (<0.001 - 0.4)	<0.001 (<0.001 - 0.4)
CAR-T CD183- CD194+ CD196- CCR10+ CM	<0.001 (<0.001 - 0.3)	<0.001 (<0.001 - 0.3)
**CAR-Tαβ$^+$ CD8$^+$**	26 (<0.001 - 1220)	54 (<0.001 - 93)
**Maturation-associated subsets**		
CAR-Tαβ$^+$ CD8$^+$ Naïve	<0.001 (<0.001 - 0.8)	<0.001 (<0.001 - 3.1)
CAR-Tαβ$^+$ CD8$^+$ CM	18 (<0.001 - 983)	34 (<0.001 - 85)
CAR-Tαβ$^+$ CD8$^+$ TM	4.3 (<0.001 - 567)	5.2 (<0.001 - 48)
CAR-Tαβ$^+$ CD8$^+$ EM	0.3 (<0.001 - 105)	0.4 (<0.001 - 32)
CAR-Tαβ$^+$ CD8$^+$ EE	<0.001 (<0.001 - 14)	<0.001 (<0.001 - 7)
CAR-Tαβ$^+$ CD8$^+$ TE	<0.001 (<0.001 - 5.8)	<0.001 (<0.001 - 1.4)
**CAR-Tγδ$^+$**	<0.001 (<0.001 - 13)	0.1 (<0.001 - 5.7)
**Maturation-associated subsets**		
CAR-Tγδ$^+$ CM	<0.001 (<0.001 - 9.8)	<0.001 (<0.001 - 2.8)
CAR-Tγδ$^+$ TM	<0.001 (<0.001 - 3.2)	<0.001 (<0.001 - 0.7)
CAR-Tγδ$^+$ EM	<0.001 (<0.001 - 3.7)	<0.001 (<0.001 - 2.2)
CAR-Tγδ$^+$ EE	<0.001 (<0.001 - 0.05)	<0.001 (<0.001 - 0.1)
CAR-Tγδ$^+$ TE	<0.001 (<0.001 - 0.3)	<0.001 (<0.001 - 0.04)
**CAR-Tαβ$^+$ DN**	<0.001 (<0.001 - 45)	<0.001 (<0.001 - 24)

Results expressed as median number of cells/µL (range) and median percentage (range) from all anti-CD19 CAR-T cells. CM, central memory; DN, double negative (CD4- CD8-/+); EE, early effector; EM, effector memory; NS, no statistically significant differences detected (p>0.05); TE, terminal effector; TFH, T follicular helper cells; Th, T helper cells; TM, transitional memory; Tregs, T regulatory cells.

Supplementary Table S4: Differences in the composition of the tisa-cel and axi-cel anti-CD19 CAR-T cells at the CAR-T peak in blood of DLBCL patients (N=58).

	**tisa-cel** N = 25	**axi-cel** N = 33	***p* value**
**CAR-T cells, cells/µL**	34 (0.5 - 568)	113 (2.3 - 1771)	*0.01*
**CAR-Tαβ$^+$ CD4$^+$, cells/µL**	14 (0.02 - 433)	42 (0.5 - 631)	*0.003*
**Maturation-associated subsets**			
CAR-Tαβ$^+$ CD4$^+$ CM, cells/µL	6.7 (<0.001 - 309)	23 (0.4 - 478)	*0.006*
CAR-Tαβ$^+$ CD4$^+$ TM, cells/µL	1.1 (<0.001 - 117)	3.1 (<0.001 - 87)	*NS*
CAR-Tαβ$^+$ CD4$^+$ EM, cells/µL	1.5 (<0.001 - 37)	4.4 (<0.001 - 115)	*NS*
CAR-Tαβ$^+$ CD4$^+$ TE, cells/µL	<0.001 (<0.001 - 0.2)	<0.001 (<0.001 - 1.1)	*NS*
**Functional (and maturation)-associated subsets**			
CAR-TFH, cells/µL	0.01 (<0.001 - 2)	0.2 (<0.001 - 9.5)	*NS*
CAR-TFH Regulatory, cells/µL	<0.001 (<0.001 - 0.2)	<0.001 (<0.001 - 0.4)	*NS*
CAR-TFH Th1 Like, cells/µL	<0.001 (<0.001 - 0.4)	0.05 (<0.001 - 5.9)	*0.05*
CAR-TFH Th2 Like, cells/µL	<0.001 (<0.001 - 0.08)	<0.001 (<0.001 - 0.06)	*NS*
CAR-TFH Th1/Th2 Like, cells/µL	<0.001 (<0.001 - 0.4)	<0.001 (<0.001 - 6.4)	*NS*
CAR-TFH Th17 Like, cells/µL	<0.001 (<0.001 - 0.007)	<0.001 (<0.001 - 0.05)	*NS*
CAR-TFH Th1/Th17 Like, cells/µL	<0.001 (<0.001 - 0.02)	<0.001 (<0.001 - 0.3)	*NS*
CAR-TFH CD183+ CD194+ CD196+ CCR10-, cells/µL	<0.001 (<0.001 - 1)	<0.001 (<0.001 - 0.8)	*NS*
CAR-Tregs, cells/µL	0.4 (<0.001 - 17)	6.8 (<0.001 - 120)	*<0.001*
CAR-Tregs Th1 Like, cells/µL	0.07 (<0.001 - 11)	3.5 (<0.001 - 69)	*<0.001*
CAR-Tregs Th2 Like, cells/µL	<0.001 (<0.001 - 0.8)	<0.001 (<0.001 - 0.5)	*NS*
CAR-Tregs Th1/Th2 Like, cells/µL	0.07 (<0.001 - 3.4)	2.8 (<0.001 - 48)	*<0.001*
CAR-Tregs Th17 Like, cells/µL	<0.001 (<0.001 - 0.4)	<0.001 (<0.001 - 0.1)	*NS*
CAR-Tregs Th1/Th17 Like, cells/µL	<0.001 (<0.001 - 1.3)	<0.001 (<0.001 - 1.4)	*NS*
CAR-Tregs CD183+ CD194+ CD196+ CCR10-, cells/µL	<0.001 (<0.001 - 0.5)	<0.001 (<0.001 - 3.9)	*NS*
CAR-Th1, cells/µL	5 (<0.001 - 69)	26 (0.3 - 482)	*<0.001*
CAR-Th1 CM, cells/µL	3.2 (<0.001 - 57)	15 (0.3 - 363)	*<0.001*
CAR-Th1 TM, cells/µL	0.4 (<0.001 - 38)	2 (<0.001 - 74)	*0.009*
CAR-Th1 EM, cells/µL	0.7 (<0.001 - 28)	2.4 (<0.001 - 115)	*0.01*
CAR-Th1 TE, cells/µL	<0.001 (<0.001 - 0.2)	<0.001 (<0.001 - 1)	*NS*
CAR-Th2, cells/µL	0.3 (<0.001 - 18)	<0.001 (<0.001 - 7.4)	*0.02*
CAR-Th2 CM, cells/µL	0.1 (<0.001 - 14)	<0.001 (<0.001 - 0.7)	*0.02*
CAR-Th2 TM, cells/µL	<0.001 (<0.001 - 1.1)	<0.001 (<0.001 - 0.3)	*NS*
CAR-Th2 EM, cells/µL	0.04 (<0.001 - 2.9)	<0.001 (<0.001 - 6.7)	*0.01*
CAR-Th2 TE, cells/µL	<0.001 (<0.001 - 0.008)	<0.001 (<0.001 - 0.03)	*NS*
CAR-Th1/Th2, cells/µL	2.2 (<0.001 - 354)	7.8 (<0.001 - 187)	*NS*
CAR-Th1/Th2 CM, cells/µL	1.4 (<0.001 - 276)	5.9 (<0.001 - 184)	*NS*
CAR-Th1/Th2 TM, cells/µL	0.2 (<0.001 - 78)	0.4 (<0.001 - 18)	*NS*
CAR-Th1/Th2 EM, cells/µL	0.4 (<0.001 - 18)	0.3 (<0.001 - 5.8)	*NS*
CAR-Th1/Th2 TE, cells/µL	<0.001 (<0.001 - 0.008)	<0.001 (<0.001 - 0.05)	*NS*
CAR-Th17, cells/µL	0.1 (<0.001 - 10)	<0.001 (<0.001 - 2.1)	*0.03*
CAR-Th17 CM, cells/µL	<0.001 (<0.001 - 4.2)	<0.001 (<0.001 - 0.6)	*NS*
CAR-Th17 TM, cells/µL	<0.001 (<0.001 - 0.5)	<0.001 (<0.001 - 0.4)	*NS*
CAR-Th17 EM, cells/µL	<0.001 (<0.001 - 5.8)	<0.001 (<0.001 - 1.1)	*0.009*
CAR-Th1/Th17, cells/µL	0.4 (<0.001 - 7.8)	0.2 (<0.001 - 21)	*NS*
CAR-Th1/Th17 CM, cells/µL	0.1 (<0.001 - 1.1)	0.1 (<0.001 - 12)	*NS*
CAR-Th1/Th17 TM, cells/µL	0.1 (<0.001 - 0.9)	<0.001 (<0.001 - 6.9)	*NS*
CAR-Th1/Th17 EM, cells/µL	0.1 (<0.001 - 5.8)	<0.001 (<0.001 - 4.1)	*NS*
CAR-Th22, cells/µL	<0.001 (<0.001 - 0.2)	<0.001 (<0.001 - 0.09)	*NS*
CAR-Th22 CM, cells/µL	<0.001 (<0.001 - 0.07)	<0.001 (<0.001 - 0.01)	*NS*
CAR-Th22 EM, cells/µL	<0.001 (<0.001 - 0.1)	<0.001 (<0.001 - 0.04)	*NS*
CAR-T CD183+ CD194+ CD196+ CCR10+, cells/µL	<0.001 (<0.001 - 0.5)	<0.001 (<0.001 - 0.1)	*NS*
CAR-T CD183+ CD194+ CD196+ CCR10+ CM, cells/µL	<0.001 (<0.001 - 0.3)	<0.001 (<0.001 - 0.07)	*NS*
CAR-T CD183+ CD194+ CD196+ CCR10+ TM, cells/µL	<0.001 (<0.001 - 0.07)	<0.001 (<0.001 - 0.09)	*NS*
CAR-T CD183+ CD194+ CD196+ CCR10+ EM, cells/µL	<0.001 (<0.001 - 0.2)	<0.001 (<0.001 - 0.01)	*NS*
CAR-T CD183+ CD194+ CD196+ CCR10-, cells/µL	0.7 (<0.001 - 28)	0.1 (<0.001 - 19)	*NS*
CAR-T CD183+ CD194+ CD196+ CCR10- CM, cells/µL	0.3 (<0.001 - 10)	0.1 (<0.001 - 16)	*NS*
CAR-T CD183+ CD194+ CD196+ CCR10- TM, cells/µL	5.2 (<0.001 - 131)	0.1 (<0.001 - 19)	*<0.001*
CAR-T CD183+ CD194+ CD196+ CCR10- EM, cells/µL	0.3 (<0.001 - 14)	0.009 (<0.001 - 1.8)	*0.004*
CAR-T CD183+ CD194+ CD196+ CCR10- TE, cells/µL	<0.001 (<0.001 - 0.02)	<0.001 (<0.001 - 0.01)	*NS*
CAR-T CD183+ CD194+ CD196- CCR10+, cells/µL	<0.001 (<0.001 - 0.4)	<0.001 (<0.001 - 0.8)	*NS*
CAR-T CD183+ CD194+ CD196- CCR10+ CM, cells/µL	<0.001 (<0.001 - 0.2)	<0.001 (<0.001 - 0.7)	*NS*
CAR-T CD183+ CD194+ CD196- CCR10+ TM, cells/µL	<0.001 (<0.001 - 0.1)	<0.001 (<0.001 - 0.1)	*NS*
CAR-T CD183+ CD194+ CD196- CCR10+ EM, cells/µL	<0.001 (<0.001 - 0.08)	<0.001 (<0.001 - 0.08)	*NS*
CAR-T CD183- CD194+ CD196- CCR10+, cells/µL	<0.001 (<0.001 - 0.4)	<0.001 (<0.001 - 0.02)	*NS*
CAR-T CD183- CD194+ CD196- CCR10+ CM, cells/µL	<0.001 (<0.001 - 0.3)	<0.001 (<0.001 - 0.005)	*NS*
**CAR-Tαβ$^+$ CD8$^+$, cells/µL**	19 (0.4 - 482)	47 (<0.001 - 1220)	*NS*
**Maturation-associated subsets**			
CAR-Tαβ$^+$ CD8$^+$ Naïve, cells/µL	<0.001 (<0.001 - 0.3)	<0.001 (<0.001 - 0.8)	*NS*
CAR-Tαβ$^+$ CD8$^+$ CM, cells/µL	13 (0.3 - 478)	33 (<0.001 - 983)	*NS*
CAR-Tαβ$^+$ CD8$^+$ TM, cells/µL	2.4 (<0.001 - 119)	6.3 (<0.001 - 567)	*NS*
CAR-Tαβ$^+$ CD8$^+$ EM, cells/µL	0.5 (<0.001 - 52)	0.3 (<0.001 - 105)	*NS*
CAR-Tαβ$^+$ CD8$^+$ EE, cells/µL	<0.001 (<0.001 - 14)	<0.001 (<0.001 - 0.6)	*NS*
CAR-Tαβ$^+$ CD8$^+$ TE, cells/µL	<0.001 (<0.001 - 5.8)	<0.001 (<0.001 - 0.2)	*NS*
**CAR-Tγδ$^+$, cells/µL**	<0.001 (<0.001 - 4.3)	<0.001 (<0.001 - 13)	*NS*
**Maturation-associated subsets**			
CAR-Tγδ$^+$ CM, cells/µL	<0.001 (<0.001 - 2)	<0.001 (<0.001 - 9.8)	*NS*
CAR-Tγδ$^+$ TM, cells/µL	<0.001 (<0.001 - 1.3)	<0.001 (<0.001 - 3.2)	*NS*
CAR-Tγδ$^+$ EM, cells/µL	<0.001 (<0.001 - 1.6)	<0.001 (<0.001 - 3.7)	*NS*
CAR-Tγδ$^+$ EE, cells/µL	<0.001 (<0.001 - 0.05)	<0.001 (<0.001 - 0.05)	*NS*
CAR-Tγδ$^+$ TE, cells/µL	<0.001 (<0.001 - 0.1)	<0.001 (<0.001 - 0.3)	*NS*
**CAR-Tαβ$^+$ DN, cells/µL**	<0.001 (<0.001 - 45)	<0.001 (<0.001 - 15)	*NS*

Results expressed as median number of cells/µL (range). CM, central memory; DN, double negative (CD4- CD8-/+); EE, early effector; EM, effector memory; NS, no statistically significant differences detected (p>0.05); TE, terminal effector; TFH, T follicular helper cells; Th, T helper cells; TM, transitional memory; Tregs, T regulatory cells.

Supplementary Table S5: Differences in the composition of the tisa-cel and axi-cel anti-CD19 CAR-T cells at the CAR-T peak in blood of DLBCL patients (N=58).

	tisa-cel N = 25	axi-cel N = 33	*p* value
**CAR-T cells (% within total cells)**	2.3 (0.04 - 19)	14 (0.04 - 55)	*<0.001*
**CAR-Tαβ$^+$ CD4$^+$, %**	38 (0.1 - 94)	59 (16 - 100)	*NS*
**Maturation-associated subsets**			
CAR-Tαβ$^+$ CD4$^+$ CM, %	17 (<0.001 - 58)	26 (5.5 - 82)	*NS*
CAR-Tαβ$^+$ CD4$^+$ TM, %	3.2 (<0.001 - 21)	2 (<0.001 - 21)	*NS*
CAR-Tαβ$^+$ CD4$^+$ EM, %	3.9 (<0.001 - 44)	3 (<0.001 - 35)	*NS*
CAR-Tαβ$^+$ CD4$^+$ TE, %	<0.001 (<0.001 - 0.1)	<0.001 (<0.001 - 1.6)	*NS*
**Functional (and maturation)-associated subsets**			
CAR-TFH, %	0.07 (<0.001 - 2.2)	0.07 (<0.001 - 2.8)	*NS*
CAR-TFH Regulatory, %	<0.001 (<0.001 - 0.5)	<0.001 (<0.001 - 0.2)	*NS*
CAR-TFH Th1 Like, %	<0.001 (<0.001 - 2.2)	0.02 (<0.001 - 1.6)	*NS*
CAR-TFH Th2 Like, %	<0.001 (<0.001 - 0.05)	<0.001 (<0.001 - 0.1)	*NS*
CAR-TFH Th1/Th2 Like, %	<0.001 (<0.001 - 0.9)	<0.001 (<0.001 - 1.3)	*NS*
CAR-TFH Th17 Like, %	<0.001 (<0.001 - 0.05)	<0.001 (<0.001 - 0.04)	*NS*
CAR-TFH Th1/Th17 Like, %	<0.001 (<0.001 - 0.01)	<0.001 (<0.001 - 0.1)	*NS*
CAR-TFH CD183+ CD194+ CD196+ CCR10-, %	<0.001 (<0.001 - 0.3)	<0.001 (<0.001 - 0.2)	*NS*
CAR-Tregs, %	1.1 (<0.001 - 13)	4.2 (<0.001 - 68)	*0.002*
CAR-Tregs Th1 Like, %	0.07 (<0.001 - 9.4)	1.5 (<0.001 - 40)	*0.004*
CAR-Tregs Th2 Like, %	<0.001 (<0.001 - 0.5)	<0.001 (<0.001 - 0.7)	*NS*
CAR-Tregs Th1/Th2 Like, %	0.05 (<0.001 - 2.5)	2.3 (<0.001 - 35)	*<0.001*
CAR-Tregs Th17 Like, %	<0.001 (<0.001 - 0.3)	<0.001 (<0.001 - 0.1)	*NS*
CAR-Tregs Th1/Th17 Like, %	<0.001 (<0.001 - 0.6)	<0.001 (<0.001 - 0.8)	*NS*
CAR-Tregs CD183+ CD194+ CD196+ CCR10-, %	<0.001 (<0.001 - 0.7)	0.002 (<0.001 - 1.6)	*NS*
CAR-Th1, %	12 (<0.001 - 50)	21 (5.5 - 84)	*0.007*
CAR-Th1 CM, %	5.9 (<0.001 - 43)	15 (3.2 - 67)	*0.002*
CAR-Th1 TM, %	1.8 (<0.001 - 7.7)	1.5 (<0.001 - 16)	*NS*
CAR-Th1 EM, %	1.1 (<0.001 - 26)	2.3 (<0.001 - 33)	*NS*
CAR-Th1 TE, %	<0.001 (<0.001 - 0.1)	<0.001 (<0.001 - 1.5)	*NS*
CAR-Th2, %	0.3 (<0.001 - 18)	<0.001 (<0.001 - 1.2)	*0.01*
CAR-Th2 CM, %	0.1 (<0.001 - 14)	<0.001 (<0.001 - 0.6)	*0.009*
CAR-Th2 TM, %	<0.001 (<0.001 - 1.1)	<0.001 (<0.001 - 0.3)	*NS*
CAR-Th2 EM, %	0.05 (<0.001 - 3.6)	<0.001 (<0.001 - 1.1)	*0.009*
CAR-Th2 TE, %	<0.001 (<0.001 - 0.02)	<0.001 (<0.001 - 0.04)	*NS*
CAR-Th1/Th2, %	5 (<0.001 - 62)	7.3 (<0.001 - 64)	*NS*
CAR-Th1/Th2 CM, %	3.3 (<0.001 - 49)	5.6 (<0.001 - 41)	*NS*
CAR-Th1/Th2 TM, %	0.3 (<0.001 - 14)	0.2 (<0.001 - 11)	*NS*
CAR-Th1/Th2 EM, %	0.4 (<0.001 - 12)	0.3 (<0.001 - 12)	*NS*
CAR-Th1/Th2 TE, %	<0.001 (<0.001 - 0.02)	<0.001 (<0.001 - 0.1)	*NS*
CAR-Th17, %	0.1 (<0.001 - 10)	<0.001 (<0.001 - 0.4)	*0.02*
CAR-Th17 CM, %	0.005 (<0.001 - 4.2)	<0.001 (<0.001 - 0.2)	*0.05*
CAR-Th17 TM, %	<0.001 (<0.001 - 0.7)	<0.001 (<0.001 - 0.08)	*NS*
CAR-Th17 EM, %	<0.001 (<0.001 - 5.8)	<0.001 (<0.001 - 0.2)	*0.007*
CAR-Th1/Th17, %	0.6 (<0.001 - 11)	0.1 (<0.001 - 4.2)	*NS*
CAR-Th1/Th17 CM, %	0.1 (<0.001 - 2.4)	<0.001 (<0.001 - 2.1)	*0.007*
CAR-Th1/Th17 TM, %	0.1 (<0.001 - 1.3)	<0.001 (<0.001 - 1.5)	*NS*
CAR-Th1/Th17 EM, %	0.3 (<0.001 - 8.1)	<0.001 (<0.001 - 2.9)	*0.05*
CAR-Th22, %	<0.001 (<0.001 - 0.2)	<0.001 (<0.001 - 0.1)	*NS*
CAR-Th22 CM, %	<0.001 (<0.001 - 0.07)	<0.001 (<0.001 - 0.05)	*NS*
CAR-Th22 EM, %	<0.001 (<0.001 - 0.1)	<0.001 (<0.001 - 0.05)	*NS*
CAR-T CD183+ CD194+ CD196+ CCR10+, %	<0.001 (<0.001 - 4)	<0.001 (<0.001 - 0.05)	*NS*
CAR-T CD183+ CD194+ CD196+ CCR10+ CM, %	<0.001 (<0.001 - 3)	<0.001 (<0.001 - 0.04)	*NS*
CAR-T CD183+ CD194+ CD196+ CCR10+ TM, %	<0.001 (<0.001 - 0.2)	<0.001 (<0.001 - 0.01)	*NS*
CAR-T CD183+ CD194+ CD196+ CCR10+ EM, %	<0.001 (<0.001 - 1)	<0.001 (<0.001 - 0.003)	*NS*
CAR-T CD183+ CD194+ CD196+ CCR10-, %	1.8 (<0.001 - 31)	0.1 (<0.001 - 5.5)	*0.002*
CAR-T CD183+ CD194+ CD196+ CCR10- CM, %	0.6 (<0.001 - 10)	0.1 (<0.001 - 4.5)	*0.01*
CAR-T CD183+ CD194+ CD196+ CCR10- TM, %	0.1 (<0.001 - 5.3)	0.006 (<0.001 - 0.7)	*<0.001*
CAR-T CD183+ CD194+ CD196+ CCR10- EM, %	0.7 (<0.001 - 19)	0.006 (<0.001 - 0.4)	*<0.001*
CAR-T CD183+ CD194+ CD196+ CCR10- TE, %	<0.001 (<0.001 - 0.05)	<0.001 (<0.001 - 0.009)	*NS*
CAR-T CD183+ CD194+ CD196- CCR10+, %	<0.001 (<0.001 - 1.5)	<0.001 (<0.001 - 0.2)	*NS*
CAR-T CD183+ CD194+ CD196- CCR10+ CM, %	<0.001 (<0.001 - 0.8)	<0.001 (<0.001 - 0.2)	*NS*
CAR-T CD183+ CD194+ CD196- CCR10+ TM, %	<0.001 (<0.001 - 0.4)	<0.001 (<0.001 - 0.03)	*NS*
CAR-T CD183+ CD194+ CD196- CCR10+ EM, %	<0.001 (<0.001 - 0.2)	<0.001 (<0.001 - 0.02)	*NS*
CAR-T CD183- CD194+ CD196- CCR10+, %	<0.001 (<0.001 - 0.4)	<0.001 (<0.001 - 0.004)	*NS*
CAR-T CD183- CD194+ CD196- CCR10+ CM, %	<0.001 (<0.001 - 0.3)	<0.001 (<0.001 - 0.001)	*NS*
**CAR-Tαβ$^+$ CD8$^+$, %**	62 (6 - 93)	41 (<0.001 - 83)	*NS*
**Maturation-associated subsets**			
CAR-Tαβ$^+$ CD8$^+$ Naïve, %	<0.001 (<0.001 - 0.4)	<0.001 (<0.001 - 3.1)	*NS*
CAR-Tαβ$^+$ CD8$^+$ CM, %	43 (3.2 - 85)	31 (<0.001 - 69)	*NS*
CAR-Tαβ$^+$ CD8$^+$ TM, %	9.5 (<0.001 - 33)	4.5 (<0.001 - 48)	*NS*
CAR-Tαβ$^+$ CD8$^+$ EM, %	0.6 (<0.001 - 28)	0.3 (<0.001 - 32)	*NS*
CAR-Tαβ$^+$ CD8$^+$ EE, %	<0.001 (<0.001 - 7)	<0.001 (<0.001 - 1.6)	*NS*
CAR-Tαβ$^+$ CD8$^+$ TE, %	<0.001 (<0.001 - 1.4)	<0.001 (<0.001 - 0.1)	*NS*
**CAR-Tγδ$^+$, %**	<0.001 (<0.001 - 5.7)	0.1 (<0.001 - 1.9)	*NS*
**Maturation-associated subsets**			
CAR-Tγδ$^+$ CM, %	<0.001 (<0.001 - 2.8)	<0.001 (<0.001 - 1.6)	*NS*
CAR-Tγδ$^+$ TM, %	<0.001 (<0.001 - 0.7)	<0.001 (<0.001 - 0.3)	*NS*
CAR-Tγδ$^+$ EM, %	<0.001 (<0.001 - 2.2)	<0.001 (<0.001 - 0.2)	*NS*
CAR-Tγδ$^+$ EE, %	<0.001 (<0.001 - 0.1)	<0.001 (<0.001 - 0.02)	*NS*
CAR-Tγδ$^+$ TE, %	<0.001 (<0.001 - 0.04)	<0.001 (<0.001 - 0.02)	*NS*
**CAR-Tαβ$^+$ DN, %**	0.1 (<0.001 - 24)	<0.001 (<0.001 - 10)	*0.04*

Results expressed as median percent values (within total CAR-T cells) and range between brackets. CM, central memory; DN, double negative (CD4- CD8-/+); EE, early effector; EM, effector memory; NS, no statistically significant differences detected (p>0.05); TE, terminal effector; TFH, T follicular helper cells; Th, T helper cells; TM, transitional memory; Tregs, T regulatory cells.

Supplementary Table S6: Differences between distinct commercially available anti-CD19 CAR-T products in post-infusion CAR-T cell features and other immune cell kinetics and patient outcome (N=58).

	**Total** N=58	**tisa-cel** N=25	**axi-cel** N=33	***p* value**
**CAR-T cells**				
No. of CAR-T cells/μL at the CAR-T peak*	68 (0.5 - 1771)	34 (0.5 - 568)	113 (2.3 - 1771)	*0.01*
Time to reach the CAR-T peak, days*	7 (7 - 55)	14 (7 - 28)	7 (7 - 55)	*NS*
No. of anti-CD19 receptors/cells at CAR-T peak (stain index)*	8.5 (2.1 - 26)	9.8 (4.1 - 23)	8.4 (2.1 - 26)	*NS*
CAR-T cell persistence, days*	58 (7 - 1052)	29 (7 - 1052)	125 (7 - 814)	*NS*
Cumulative incidence of loss of CAR-T persistence (6 months)*	64 (50 - 75)†	72 (49 - 86)†	58 (39 - 73)†	*NS*
CAR-T cell AUC days 0 - 28, days x cells/μL*	741 (5 - 12730)	330 (5 - 8469)	987 (16 - 12730)	*0.03*
No. of CAR-T TCRγδ cells at the CAR-T peak ≥ 0.06 cells/μL	35/58 (60%)	13/25 (52%)	22/33 (67%)	*NS*
**Toxicity**				
Neurotoxicity (ICANS)	15/58 (26%)	2/25 (8%)	13/33 (39%)	*0.008*
Loss of B cell aplasia	8/58 (14%)	1/25 (4%)	7/33 (21%)	*0.06*
Time to B-cell recovery, months*	6 (2 - 48)	6	6.5 (2 - 48)	-
**Response to therapy and outcomes**				
CR after CAR-T therapy	33/58 (57%)	10/25 (40%)	23/33 (70%)	*0.02*
CR after CAR-T therapy in patients ≥60 years old	17/32 (53%)	6/19 (32%)	11/13 (85%)	*0.03*
CR after CAR-T therapy in patients <60 years old	16/26 (62%)	4/6 (67%)	12/20 (60%)	*NS*
Time to CR, days*	30 (21 - 180)	29 (27 - 99)	31 (21 - 180)	*NS*
No. of recurrences	3/33 (9%)	1/10 (10%)	2/23 (9%)	*NS*
Duration of CR, months*	8 (3 - 9)	3	8 (8 - 9)	-
Time to disease progression, days*	30 (14 - 149)	34 (17 - 149)	30 (14 - 98)	*NS*
Follow-up time, months*	24 (1 - 54)	24 (1 - 49)	34 (1 - 54)	*NS*

Results expresed as number of cases/total cases (percentages) or as *median (range) value or as †median (95% CI). AUC, area under the curve; CR, complete response; ICANS, immune effector cell-associated neurotoxicity syndrome; NS, no statistically significant differences found (p>0.05); ORR, overall response rate (calculated as complete response plus partial response).

Supplementary Table S7: Distribution of circulating anti-CD19 CAR-T cell subpopulations in blood of DLBCL patients at the CAR-T peak according to response to therapy (N=58).

	Partial responders and Non-responders N=25	Complete responders N=33	*p* value Uni-variate	*p* value Multi-variate
**CAR-T, cells/µL**	29 (0.5 - 603)	132 (5.2 - 1771)	*0.002*	
**CAR-T$\alpha\beta^+$ CD4$^+$, cells/µL**	15 (0.1 - 429)	46 (<0.001 - 622)	*0.006*	
**Maturation-associated subsets**				
CAR-T$\alpha\beta^+$ CD4$^+$ CM, cells/µL	5.3 (0.09 - 234)	25 (<0.001 - 493)	*0.002*	
CAR-T$\alpha\beta^+$ CD4$^+$ TM, cells/µL	1.1 (<0.001 - 186)	4.1 (<0.001 - 58)	*0.008*	
CAR-T$\alpha\beta^+$ CD4$^+$ EM, cells/µL	1 (<0.001 - 40)	6.4 (<0.001 - 115)	*0.009*	
CAR-T$\alpha\beta^+$ CD4$^+$ TE, cells/µL	<0.001 (<0.001 - 0.02)	<0.001 (<0.001 - 2.2)	*NS*	
**Functional (and maturation)-associated subsets**				
CAR-TFH, cells/µL	<0.001 (<0.001 - 13)	0.2 (<0.001 - 11)	*0.03*	
CAR-TFH Regulatory, cells/µL	<0.001 (<0.001 - 0.3)	<0.001 (<0.001 - 1.2)	*0.05*	
CAR-TFH Th1 Like, cells/µL	<0.001 (<0.001 - 2.2)	0.05 (<0.001 - 6.2)	*0.008*	
CAR-TFH Th2 Like, cells/µL	<0.001 (<0.001 - 0.5)	<0.001 (<0.001 - 0.2)	*NS*	
CAR-TFH Th1/Th2 Like, cells/µL	<0.001 (<0.001 - 10)	0.02 (<0.001 - 2.7)	*0.008*	
CAR-TFH Th17 Like, cells/µL	<0.001 (<0.001 - 0.1)	<0.001 (<0.001 - 0.06)	*NS*	
CAR-TFH Th1/Th17 Like, cells/µL	<0.001 (<0.001 - <0.001)	<0.001 (<0.001 - 0.6)	*NS*	
CAR-TFH CD183+ CD194+ CD196+ CCR10-, cells/µL	<0.001 (<0.001 - 0.3)	<0.001 (<0.001 - 0.6)	*NS*	
CAR-Tregs, cells/µL	3.1 (<0.001 - 59)	2.7 (<0.001 - 83)	*NS*	
CAR-Tregs Th1 Like, cells/µL	1 (<0.001 - 39)	1.1 (<0.001 - 52)	*NS*	
CAR-Tregs Th2 Like, cells/µL	<0.001 (<0.001 - 1.9)	<0.001 (<0.001 - 0.8)	*0.01*	
CAR-Tregs Th1/Th2 Like, cells/µL	0.6 (<0.001 - 43)	1.5 (<0.001 - 39)	*NS*	
CAR-Tregs Th17 Like, cells/µL	<0.001 (<0.001 - <0.001)	<0.001 (<0.001 - 0.3)	*0.02*	
CAR-Tregs Th1/Th17 Like, cells/µL	<0.001 (<0.001 - 1.9)	<0.001 (<0.001 - 1.7)	*NS*	
CAR-Tregs CD183+ CD194+ CD196+ CCR10-, cells/µL	<0.001 (<0.001 - 8.5)	0.06 (<0.001 - 4.2)	*NS*	
CAR-Th1, cells/µL	4.3 (<0.001 - 130)	22 (<0.001 - 470)	*<0.001*	
CAR-Th1 CM, cells/µL	2.6 (<0.001 - 89)	15 (<0.001 - 366)	*<0.001*	
CAR-Th1 TM, cells/µL	0.5 (<0.001 - 64)	2.9 (<0.001 - 38)	*0.003*	*0.009*
CAR-Th1 EM, cells/µL	0.4 (<0.001 - 30)	2.4 (<0.001 - 115)	*0.006*	
CAR-Th1 TE, cells/µL	<0.001 (<0.001 - <0.001)	<0.001 (<0.001 - 2)	*NS*	
CAR-Th2, cells/µL	<0.001 (<0.001 - 16)	0.07 (<0.001 - 9.8)	*NS*	
CAR-Th2 CM, cells/µL	<0.001 (<0.001 - 11)	0.04 (<0.001 - 6.3)	*0.06*	
CAR-Th2 TM, cells/µL	<0.001 (<0.001 - 1.7)	<0.001 (<0.001 - 1.1)	*NS*	
CAR-Th2 EM, cells/µL	<0.001 (<0.001 - 2.9)	<0.001 (<0.001 - 2.4)	*NS*	
CAR-Th2 TE, cells/µL	<0.001 (<0.001 - <0.001)	<0.001 (<0.001 - 0.03)	*NS*	
CAR-Th1/Th2, cells/µL	1.8 (<0.001 - 321)	9.9 (<0.001 - 157)	*0.02*	
CAR-Th1/Th2 CM, cells/µL	1.3 (<0.001 - 198)	7 (<0.001 - 149)	*0.02*	
CAR-Th1/Th2 TM, cells/µL	0.2 (<0.001 - 122)	0.6 (<0.001 - 23)	*0.05*	
CAR-Th1/Th2 EM, cells/µL	0.1 (<0.001 - 5.1)	0.4 (<0.001 - 15)	*NS*	
CAR-Th1/Th2 TE, cells/µL	<0.001 (<0.001 - <0.001)	<0.001 (<0.001 - 0.05)	*NS*	
CAR-Th17, cells/µL	<0.001 (<0.001 - 14)	<0.001 (<0.001 - 4.8)	*0.04*	
CAR-Th17 CM cells/µL	<0.001 (<0.001 - 5.3)	<0.001 (<0.001 - 1.1)	*0.03*	
CAR-Th17 TM, cells/µL	<0.001 (<0.001 - 0.6)	<0.001 (<0.001 - 1.4)	*NS*	
CAR-Th17 EM, cells/µL	<0.001 (<0.001 - 8.5)	<0.001 (<0.001 - 3.1)	*NS*	
CAR-Th1/Th17, cells/µL	<0.001 (<0.001 - 6.3)	0.5 (<0.001 - 26)	*0.05*	
CAR-Th1/Th17 CM, cells/µL	<0.001 (<0.001 - 1.2)	0.1 (<0.001 - 15)	*0.01*	
CAR-Th1/Th17 TM, cells/µL	<0.001 (<0.001 - 0.8)	<0.001 (<0.001 - 9.5)	*NS*	
CAR-Th1/Th17 EM, cells/µL	<0.001 (<0.001 - 5.4)	0.2 (<0.001 - 6.9)	*NS*	
CAR-Th22, cells/µL	<0.001 (<0.001 - 0.2)	<0.001 (<0.001 - 0.06)	*NS*	
CAR-Th22 CM, cells/µL	<0.001 (<0.001 - 0.06)	<0.001 (<0.001 - 0.06)	*NS*	
CAR-Th22 EM, cells/µL	<0.001 (<0.001 - 0.1)	<0.001 (<0.001 - <0.001)	*NS*	
CAR-T CD183+ CD194+ CD196+ CCR10+, cells/µL	<0.001 (<0.001 - 0.05)	<0.001 (<0.001 - 0.5)	*NS*	
CAR-T CD183+ CD194+ CD196+ CCR10+ CM, cells/µL	<0.001 (<0.001 - 0.04)	<0.001 (<0.001 - 0.2)	*NS*	
CAR-T CD183+ CD194+ CD196+ CCR10+ TM, cells/µL	<0.001 (<0.001 - <0.001)	<0.001 (<0.001 - 0.2)	*NS*	
CAR-T CD183+ CD194+ CD196+ CCR10+ EM, cells/µL	<0.001 (<0.001 - 0.01)	<0.001 (<0.001 - 0.2)	*NS*	
CAR-T CD183+ CD194+ CD196+ CCR10-, cells/µL	<0.001 (<0.001 - 20)	0.6 (<0.001 - 19)	*0.02*	
CAR-T CD183+ CD194+ CD196+ CCR10- CM, cells/µL	<0.001 (<0.001 - 5.7)	0.3 (<0.001 - 13)	*0.002*	
CAR-T CD183+ CD194+ CD196+ CCR10- TM, cells/µL	<0.001 (<0.001 - 98)	0.4 (<0.001 - 122)	*NS*	
CAR-T CD183+ CD194+ CD196+ CCR10- EM, cells/µL	<0.001 (<0.001 - 11)	0.1 (<0.001 - 12)	*NS*	
CAR-T CD183+ CD194+ CD196+ CCR10- TE, cells/µL	<0.001 (<0.001 - <0.001)	<0.001 (<0.001 - 0.006)	*NS*	
CAR-T CD183+ CD194+ CD196- CCR10+, cells/µL	<0.001 (<0.001 - 0.1)	<0.001 (<0.001 - 0.7)	*0.06*	
CAR-T CD183+ CD194+ CD196- CCR10+ CM, cells/µL	<0.001 (<0.001 - 0.06)	<0.001 (<0.001 - 0.5)	*0.06*	
CAR-T CD183+ CD194+ CD196- CCR10+ TM, cells/µL	<0.001 (<0.001 - 0.06)	<0.001 (<0.001 - 0.1)	*NS*	
CAR-T CD183+ CD194+ CD196- CCR10+ EM, cells/µL	<0.001 (<0.001 - <0.001)	<0.001 (<0.001 - 0.1)	*0.02*	
CAR-T CD183- CD194+ CD196- CCR10+, cells/µL	<0.001 (<0.001 - 0.4)	<0.001 (<0.001 - 0.02)	*NS*	
CAR-T CD183- CD194+ CD196- CCR10+ CM, cells/µL	<0.001 (<0.001 - 0.3)	<0.001 (<0.001 - 0.005)	*NS*	
**CAR-T$\alpha\beta^+$ CD8$^+$, cells/µL**	9.2 (<0.001 - 501)	53 (0.4 - 1220)	*0.001*	
**Maturation-associated subsets**				
CAR-T$\alpha\beta^+$ CD8$^+$ Naïve, cells/µL	<0.001 (<0.001 - <0.001)	<0.001 (<0.001 - 1)	*NS*	
CAR-T$\alpha\beta^+$ CD8$^+$ CM, cells/µL	4.4 (<0.001 - 474)	33 (0.4 - 997)	*0.002*	
CAR-T$\alpha\beta^+$ CD8$^+$ TM, cells/µL	1 (<0.001 - 292)	8.3 (<0.001 - 553)	*0.006*	
CAR-T$\alpha\beta^+$ CD8$^+$ EM, cells/µL	0.1 (<0.001 - 74)	0.3 (<0.001 - 127)	*0.06*	
CAR-T$\alpha\beta^+$ CD8$^+$ EE, cells/µL	<0.001 (<0.001 - 23)	<0.001 (<0.001 - 0.6)	*NS*	
CAR-T$\alpha\beta^+$ CD8$^+$ TE, cells/µL	<0.001 (<0.001 - 1.9)	<0.001 (<0.001 - 0.8)	*NS*	
**CAR-T$\gamma\delta^+$, cells/µL**	<0.001 (<0.001 - 6)	0.2 (<0.001 - 13)	*<0.001*	*0.001*
**Maturation-associated subsets**				
CAR-T$\gamma\delta^+$ CM, cells/µL	<0.001 (<0.001 - 2.1)	0.2 (<0.001 - 9.8)	*<0.001*	
CAR-T$\gamma\delta^+$ TM, cells/µL	<0.001 (<0.001 - 2.3)	0.006 (<0.001 - 3.2)	*0.002*	
CAR-T$\gamma\delta^+$ EM, cells/µL	<0.001 (<0.001 - 2.2)	<0.001 (<0.001 - 3.7)	*0.01*	
CAR-T$\gamma\delta^+$ EE, cells/µL	<0.001 (<0.001 - 0.03)	<0.001 (<0.001 - 0.06)	*NS*	
CAR-T$\gamma\delta^+$ TE, cells/µL	<0.001 (<0.001 - 0.006)	<0.001 (<0.001 - 0.3)	*NS*	
**CAR-T$\alpha\beta^+$ DN, cells/µL**	<0.001 (<0.001 - 49)	<0.001 (<0.001 - 15)	*NS*	

Results expressed as median number of cells/µL (range). CM, central memory; DN, double negative (CD4$^-$ CD8$^{-/+}$); EE, early effector; EM, effector memory; NS, no statistically significant differences detected (p>0.05); TE, terminal effector; TFH, T follicular helper cells; Th, T helper cells; TM, transitional memory; Tregs, T regulatory cells.

Supplementary Table S8: Distribution of circulating anti-CD19 CAR-T cells subpopulations in blood of DLBCL patients at the CAR-T peak according to response to therapy (N=58).

	**Partial responders/No-responders**<br><br>**N=25**	**Complete responders**<br><br>**N=33**	***p* value**<br>Uni-variate	***p* value**<br>Multi-variate
**CAR-T cells (% within total cells)**	1.1 (0.04 - 19)	5.8 (0.3 - 55)	*0.004*	*0.005*
**CAR-T TCRαβ^{+} CD4^{+}, %**	59 (9 - 100)	42 (<0.001 - 93)	*NS*	
**Maturation-associated subsets**				
CAR-Tαβ^{+} CD4^{+} CM, %	17 (2.6 - 96)	27 (<0.001 - 68)	*NS*	
CAR-Tαβ^{+} CD4^{+} TM, %	3.5 (<0.001 - 33)	4.1 (<0.001 - 25)	*NS*	
CAR-Tαβ^{+} CD4^{+} EM, %	2.3 (<0.001 - 44)	3.6 (<0.001 - 39)	*NS*	
CAR-Tαβ^{+} CD4^{+} TE, %	<0.001 (<0.001 - 0.02)	<0.001 (<0.001 - 1.6)	*NS*	
**Functional (and maturation)-associated subsets**				
CAR-TFH, %	<0.001 (<0.001 - 2.7)	0.08 (<0.001 - 3.6)	*0.06*	
CAR-TFH Regulatory, %	<0.001 (<0.001 - 0.2)	<0.001 (<0.001 - 0.8)	*0.05*	
CAR-TFH Th1 Like, %	<0.001 (<0.001 - 2.2)	0.01 (<0.001 - 1.9)	*0.01*	
CAR-TFH Th2 Like, %	<0.001 (<0.001 - 0.6)	<0.001 (<0.001 - 0.1)	*NS*	
CAR-TFH Th1/Th2 Like, %	<0.001 (<0.001 - 2.2)	0.005 (<0.001 - 1.4)	*0.08*	
CAR-TFH Th17 Like, %	<0.001 (<0.001 - 0.1)	<0.001 (<0.001 - 0.1)	*NS*	
CAR-TFH Th1/Th17 Like, %	<0.001 (<0.001 - <0.001)	<0.001 (<0.001 - 0.2)	*NS*	
CAR-TFH CD183+ CD194+ CD196+ CCR10-, %	<0.001 (<0.001 - 0.3)	<0.001 (<0.001 - 0.2)	*0.03*	
CAR-Tregs, %	6 (<0.001 - 68)	3.4 (<0.001 - 26)	*NS*	
CAR-Tregs Th1 Like, %	2.6 (<0.001 - 33)	1.1 (<0.001 - 15)	*NS*	
CAR-Tregs Th2 Like, %	<0.001 (<0.001 - 0.3)	<0.001 (<0.001 - 0.7)	*0.009*	
CAR-Tregs Th1/Th12 Like, %	0.6 (<0.001 - 35)	1 (<0.001 - 19)	*NS*	
CAR-Tregs Th17 Like, %	<0.001 (<0.001 - <0.001)	<0.001 (<0.001 - 0.1)	*0.02*	
CAR-Tregs Th1/Th17 Like, %	<0.001 (<0.001 - 1.3)	<0.001 (<0.001 - 0.8)	*NS*	
CAR-Tregs CD183+ CD194+ CD196+ CCR10-, %	<0.001 (<0.001 - 4.5)	0.1 (<0.001 - 1.6)	*NS*	
CAR-Th1, %	17 (<0.001 - 60)	20 (<0.001 - 84)	*NS*	
CAR-Th1 CM, %	10 (<0.001 - 58)	12 (<0.001 - 67)	*NS*	
CAR-Th1 TM, %	2 (<0.001 - 17)	1.6 (<0.001 - 20)	*NS*	
CAR-Th1 EM, %	1.4 (<0.001 - 26)	2.4 (<0.001 - 33)	*NS*	
CAR-Th1 TE, %	<0.001 (<0.001 - <0.001)	<0.001 (<0.001 - 1.5)	*NS*	
CAR-Th2, %	<0.001 (<0.001 - 16)	0.02 (<0.001 - 8.5)	*NS*	
CAR-Th2 CM, %	<0.001 (<0.001 - 11)	0.009 (<0.001 - 7.7)	*NS*	
CAR-Th2 TM, %	<0.001 (<0.001 - 1.7)	<0.001 (<0.001 - 0.7)	*NS*	
CAR-Th2 EM, %	<0.001 (<0.001 - 3.6)	<0.001 (<0.001 - 1.6)	*NS*	
CAR-Th2 TE, %	<0.001 (<0.001 - <0.001)	<0.001 (<0.001 - 0.04)	*NS*	
CAR-Th1/Th2, %	3.1 (<0.001 - 64)	8.2 (<0.001 - 56)	*NS*	
CAR-Th1/Th2 CM, %	1.7 (<0.001 - 41)	6.3 (<0.001 - 43)	*NS*	
CAR-Th1/Th2 TM, %	0.3 (<0.001 - 21)	0.7 (<0.001 - 6.8)	*NS*	
CAR-Th1/Th2 EM, %	0.2 (<0.001 - 12)	0.4 (<0.001 - 11)	*NS*	
CAR-Th1/Th2 TE, %	<0.001 (<0.001 - <0.001)	<0.001 (<0.001 - 0.06)	*NS*	
CAR-Th17, %	<0.001 (<0.001 - 15)	<0.001 (<0.001 - 6.7)	*0.05*	
CAR-Th17 CM, %	<0.001 (<0.001 - 5.3)	<0.001 (<0.001 - 0.9)	*0.04*	
CAR-Th17 TM, %	<0.001 (<0.001 - 0.6)	<0.001 (<0.001 - 1.4)	*NS*	
CAR-Th17 EM, %	<0.001 (<0.001 - 8.6)	<0.001 (<0.001 - 4.4)	*NS*	
CAR-Th1/Th17, %	<0.001 (<0.001 - 9.2)	0.3 (<0.001 - 12)	*NS*	
CAR-Th1/Th17 CM, %	<0.001 (<0.001 - 2.4)	0.1 (<0.001 - 2.5)	*NS*	
CAR-Th1/Th17 TM, %	<0.001 (<0.001 - 0.8)	<0.001 (<0.001 - 1.6)	*NS*	
CAR-Th1/Th17 EM, %	<0.001 (<0.001 - 6.1)	0.1 (<0.001 - 9)	*NS*	
CAR-Th22, %	<0.001 (<0.001 - 0.2)	<0.001 (<0.001 - 0.02)	*NS*	
CAR-Th22 CM, %	<0.001 (<0.001 - 0.06)	<0.001 (<0.001 - 0.02)	*NS*	
CAR-Th22 EM, %	<0.001 (<0.001 - 0.1)	<0.001 (<0.001 - <0.001)	*NS*	
CAR-T CD183+ CD194+ CD196+ CCR10+, %	<0.001 (<0.001 - 4)	<0.001 (<0.001 - 0.6)	*NS*	
CAR-T CD183+ CD194+ CD196+ CCR10+ CM, %	<0.001 (<0.001 - 3)	<0.001 (<0.001 - 0.3)	*NS*	
CAR-T CD183+ CD194+ CD196+ CCR10+ TM, %	<0.001 (<0.001 - <0.001)	<0.001 (<0.001 - 0.2)	*NS*	
CAR-T CD183+ CD194+ CD196+ CCR10+ EM, %	<0.001 (<0.001 - 1)	<0.001 (<0.001 - 0.3)	*NS*	
CAR-T CD183+ CD194+ CD196+ CCR10-, %	<0.001 (<0.001 - 20)	0.4 (<0.001 - 26)	*0.03*	
CAR-T CD183+ CD194+ CD196+ CCR10- CM, %	<0.001 (<0.001 - 5.8)	0.2 (<0.001 - 5.9)	*0.009*	
CAR-T CD183+ CD194+ CD196+ CCR10- TM, %	<0.001 (<0.001 - 3.5)	<0.001 (<0.001 - 4.9)	*NS*	
CAR-T CD183+ CD194+ CD196+ CCR10- EM, %	<0.001 (<0.001 - 11)	<0.001 (<0.001 - 17)	*NS*	
CAR-T CD183+ CD194+ CD196+ CCR10- TE, %	<0.001 (<0.001 - <0.001)	<0.001 (<0.001 - 0.009)	*NS*	
CAR-T CD183+ CD194+ CD196- CCR10+, %	<0.001 (<0.001 - 0.1)	<0.001 (<0.001 - 0.4)	*NS*	
CAR-T CD183+ CD194+ CD196- CCR10+ CM, %	<0.001 (<0.001 - 0.06)	<0.001 (<0.001 - 0.3)	*NS*	
CAR-T CD183+ CD194+ CD196- CCR10+ TM, %	<0.001 (<0.001 - 0.06)	<0.001 (<0.001 - 0.1)	*NS*	
CAR-T CD183+ CD194+ CD196- CCR10+ EM, %	<0.001 (<0.001 - <0.001)	<0.001 (<0.001 - 0.1)	*0.02*	
CAR-T CD183- CD194+ CD196- CCR10+, %	<0.001 (<0.001 - 0.4)	<0.001 (<0.001 - 0.003)	*NS*	
CAR-T CD183- CD194+ CD196- CCR10+ CM, %	<0.001 (<0.001 - 0.3)	<0.001 (<0.001 - 0.001)	*NS*	
**CAR-Tαβ^{+} CD8^{+}, %**	39 (<0.001 - 90)	57 (6.9 - 93)	*NS*	
**Maturation-associated subsets**				
CAR-Tαβ^{+} CD8^{+} Naïve, %	<0.001 (<0.001 - <0.001)	<0.001 (<0.001 - 0.4)	*NS*	
CAR-Tαβ^{+} CD8^{+} CM, %	30 (<0.001 - 84)	37 (4.5 - 69)	*NS*	
CAR-Tαβ^{+} CD8^{+} TM, %	6.1 (<0.001 - 48)	6.6 (<0.001 - 48)	*NS*	
CAR-Tαβ^{+} CD8^{+} EM, %	0.1 (<0.001 - 13)	0.5 (<0.001 - 33)	*NS*	
CAR-Tαβ^{+} CD8^{+} EE, %	<0.001 (<0.001 - 20)	<0.001 (<0.001 - 1.6)	*0.05*	
CAR-Tαβ^{+} CD8^{+} TE, %	<0.001 (<0.001 - 1.6)	<0.001 (<0.001 - 1.1)	*NS*	
**CAR-Tγδ^{+}, %**	<0.001 (<0.001 - 1)	0.2 (<0.001 - 5.7)	*<0.001*	*0.003*
**Maturation-associated subsets**				
CAR-Tγδ^{+} CM, %	<0.001 (<0.001 - 0.3)	0.1 (<0.001 - 2.3)	*<0.001*	
CAR-Tγδ^{+} TM, %	<0.001 (<0.001 - 0.4)	0.003 (<0.001 - 1.1)	*0.003*	
CAR-Tγδ^{+} EM, %	<0.001 (<0.001 - 0.4)	<0.001 (<0.001 - 2.2)	*0.04*	
CAR-Tγδ^{+} EE, %	<0.001 (<0.001 - 0.005)	<0.001 (<0.001 - 0.04)	*NS*	
CAR-Tγδ^{+} TE, %	<0.001 (<0.001 - 0.02)	<0.001 (<0.001 - 0.03)	*NS*	
**CAR-Tαβ^{+} DN, %**	<0.001 (<0.001 - 24)	<0.001 (<0.001 - 20)	*NS*	

Results expressed as median percent (within total CAR-T cells) and range between brackets. CM, central memory; DN, double negative (CD4- CD8-/+); EE, early effector; EM, effector memory; NS, no statistically significant differences detected (p>0.05); TE, terminal effector; TFH, T follicular helper cells; Th, T helper cells; TM, transitional memory; Tregs, T regulatory cells.

SUPPLEMENTARY FIGURE LEGENDS

Figure S1: Anti CD19 CAR-T cells kinetic and composition at the CAR-T peak in blood of DLBCL patients (N=58). In panel A, colored dots illustrate the kinetics of circulating CAR-T cells (per microliter) in blood of each individual patient at defined time-points during follow-up. In panel B, the composition of CAR-T cells at the CAR-T peak per microliter is represented by color-coded box plots for the distinct CAR-T cell populations identified: blue (CAR-TCD4^{+} cell populations, except Tregs), yellow (Tregs), pink (CAR-TCD8^{+} cell populations) and violet (CAR-T$\gamma\delta^{+}$). Box plots extend from the 25th to the 75th percentiles, the line in the middle represents median values and vertical lines correspond to the minimum and maximum values. D, day; M, month; Y, year; TFH, T follicular helper cells; Th, T helper cells; Tregs, T regulatory cells; CM, central memory; TM, transitional memory; EM, effector memory; TE, terminal effector.

Figure S2: Differences in the kinetics and composition of distinct commercial anti -CD19 CAR-T cell products administered in this study to DLBCL patients (n=58). (A) Comparison of the kinetics of tisa-cel vs. axi-cel anti-CD19 CAR-T cells in blood of DLBCL patients receiving each of the two CAR-T cell products at different time points after infusion. Comparison at the CAR-T peak between (B) the number of anti-CD19 CAR-T cells circulating in blood (C) the composition - in absolute cell counts (left panels) and in relative numbers (right panels) -, and (D) the stain index for the CAR receptor in DLBCL patients treated with different commercially available CAR-T cell products (tisa-cel vs. axi-cel). Tisa-cel, tisagenlecleucel; axi-cel, axicabtagene ciloleucel; D, day; M, month; Y, year; CM, central memory; TM, transitional memory; EM, effector memory; Th, T helper cells; Tregs, T regulatory cells; DN, double negative (CD4- CD8-).

Figure S3: Correlation between the number of circulating anti CD19 CAR-T cells and both normal B lymphocytes and lymphoma cells in blood of DLBCL patients after CAR-T cell infusion (n=840 samples from 58 patients). Panels (A) and (B) show the correlation between the number of circulating anti-CD19 CAR-T cells and that of both lymphoma B cells (A) and normal B lymphocytes (B) at different (color-coded) time points evaluated after CAR-T cell infusion. In panels C and D, the percentage of patients with circulating lymphoma cells (C) and normal B lymphocytes (D) coexisting or not with anti-CD19 CAR-T cells in blood at different (post-infusion) time points during follow up are shown. In panels E and F, the differences between patients with transient emergence of B lymphocytes (panel E) and patients who lost the B-cell aplasia (panel F) at different (post-infusion) time points during follow-up, are shown. Aph., aphaeresis; pre-inf., pre-infusion; D, day; M, month; Y, year.

Figure S4: Univariate and multivariate analysis of the prognostic impact clinical and biological features of the disease and CAR-T cell-associated parameters on achieving a complete response (N=58). Results expressed as number of cases/total cases or as *median (range) value. aHSCT, autologous hematopoietic stem cell transplant; IPI, international prognostic index; LDH, lactate dehydrogenase; MTV, metabolic tumor volume; TLG, total lesion glycolysis; CNS, central nervous system; AUC, area under the curve; tisa-cel, tisagenlecleucel; axi-cel, axicabtagene ciloleucel; CM, central memory; TM, transitional memory; EM, effector memory; TFH, T follicular helper cells; Th, T helper cells; NS, no statistically significant differences found ($p>0.05$).

Figure S5: Swimmer plot summarizing the outcomes of individual DLBCL patients treated with anti-CD19 CAR-T cells (n=58). For each individual patient defined by an ID code (number), grey bars represent the time lapse from CAR-T cell infusion to the last follow-up or death (+), while blue bars represent the follow-up time during which circulating CAR-T cells were detected in blood.

Figure S6: Clinical and laboratory patient features and blood circulating total anti-CD19 CAR-T cells, and their subsets counts with a prognostic impact on progression-free survival of DLBCL patients (n=58). LymDep, lymphodepletion; MTV, metabolic tumor volume; TLG, total lesion glycolysis; LDH, lactate dehydrogenase; AUC0-28, area under curve; CM, central memory; TM, transitional memory; EM, effector memory; TFH, T follicular helper cells; Th, T helper cells.

Figure S7: Clinical and laboratory patient features and blood circulating total anti-CD19 CAR-T cell and their subset counts with a prognostic impact on overall survival of DLBCL patients (n=58). LymDep, lymphodepletion; MTV, metabolic tumor volume; TLG, total lesion glycolysis; LDH, lactate dehydrogenase; AUC0-28, area under curve; CM, central memory; TM, transitional memory; EM, effector memory; TFH, T follicular helper cells; Th, T helper cells.

Figure S1

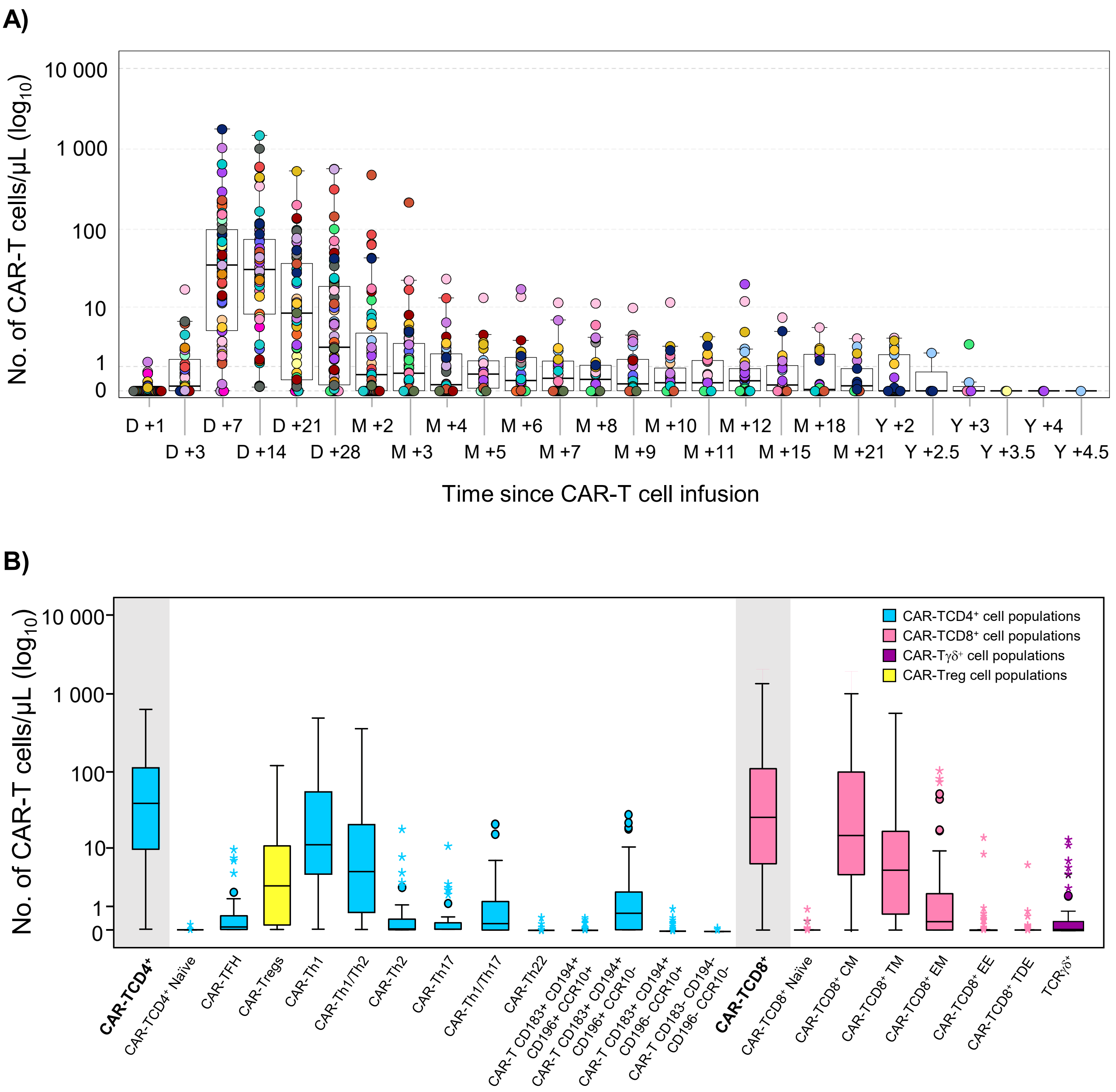
A)
No. of CAR-T cells/µL (log10)
10 000
1 000
100
10
1
0
D +1
D +3
D +7
D +14
D +21
D +28
M +2
M +3
M +4
M +5
M +6
M +7
M +8
M +9
M +10
M +11
M +12
M +15
M +18
M +21
Y +2
Y +2.5
Y +3
Y +3.5
Y +4
Y +4.5
Time since CAR-T cell infusion
B)
No. of CAR-T cells/µL (log10)
10 000
1 000
100
10
1
0
CAR-TCD4⁺ cell populations
CAR-TCD8⁺ cell populations
CAR-Tγδ⁺ cell populations
CAR-Treg cell populations
CAR-TCD4⁺
CAR-TCD4⁺ Naïve
CAR-TFH
CAR-Tregs
CAR-Th1
CAR-Th1/Th2
CAR-Th2
CAR-Th17
CAR-Th1/Th17
CAR-Th22
CAR-T CD183+ CD194+ CD196+ CCR10+
CAR-T CD183+ CD194+ CD196+ CCR10-
CAR-T CD183+ CD194+ CD196- CCR10+
CAR-T CD183- CD194- CD196- CCR10-
CAR-TCD8⁺
CAR-TCD8⁺ Naïve
CAR-TCD8⁺ CM
CAR-TCD8⁺ TM
CAR-TCD8⁺ EM
CAR-TCD8⁺ EE
CAR-TCD8⁺ TDE
TCRγδ⁺

Figure S2

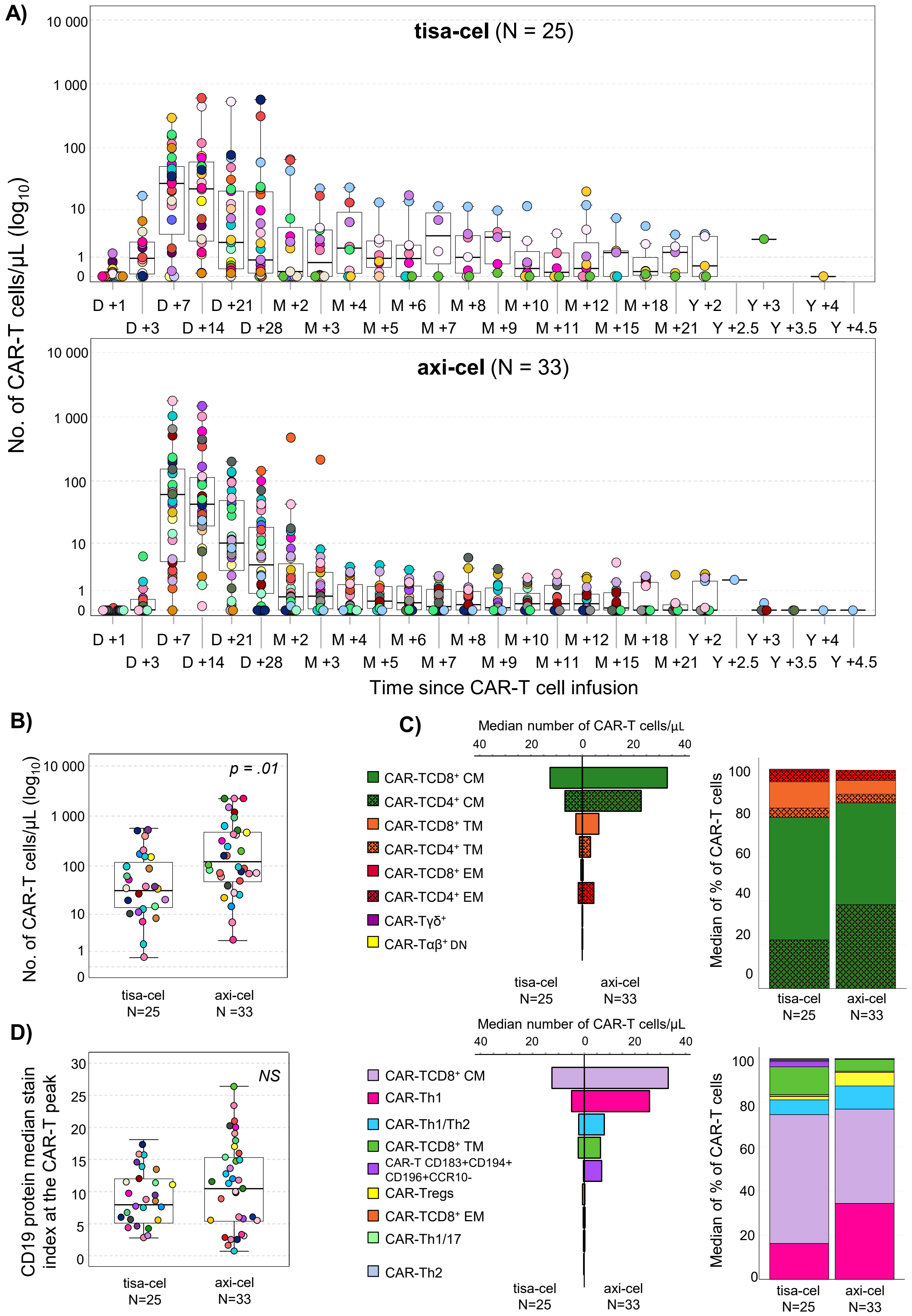
A)
tisa-cel (N = 25)
axi-cel (N = 33)
No. of CAR-T cells/µL (log10)
10 000
1 000
100
10
1
0
D +1
D +3
D +7
D +14
D +21
D +28
M +2
M +3
M +4
M +5
M +6
M +7
M +8
M +9
M +10
M +11
M +12
M +15
M +18
M +21
Y +2
Y +2.5
Y +3
Y +3.5
Y +4
Y +4.5
Time since CAR-T cell infusion
B)
p = .01
No. of CAR-T cells/µL (log10)
tisa-cel
N=25
axi-cel
N =33
C)
Median number of CAR-T cells/µL
40
20
0
20
40
CAR-TCD8+ CM
CAR-TCD4+ CM
CAR-TCD8+ TM
CAR-TCD4+ TM
CAR-TCD8+ EM
CAR-TCD4+ EM
CAR-Tγδ+
CAR-Tαβ+ DN
tisa-cel
N=25
axi-cel
N=33
Median of % of CAR-T cells
100
80
60
40
20
0
CAR-TCD8+ CM
CAR-Th1
CAR-Th1/Th2
CAR-TCD8+ TM
CAR-T CD183+CD194+
CD196+CCR10-
CAR-Tregs
CAR-TCD8+ EM
CAR-Th1/17
CAR-Th2
D)
NS
CD19 protein median stain
index at the CAR-T peak
30
25
20
15
10
5
0
tisa-cel
N=25
axi-cel
N=33

Figure S3

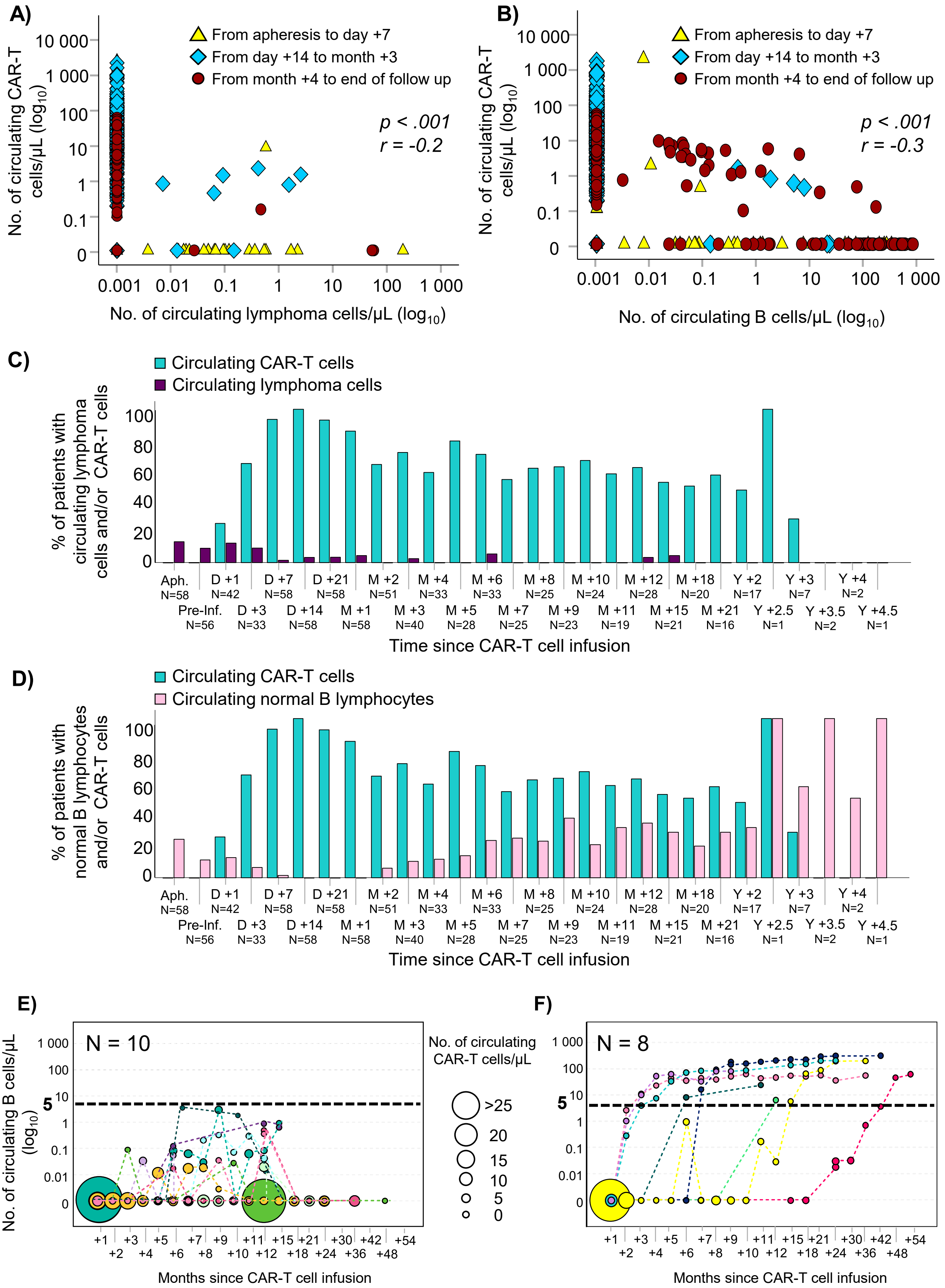

A)
From apheresis to day +7
From day +14 to month +3
From month +4 to end of follow up
p < .001
r = -0.2
No. of circulating CAR-T cells/µL (log10)
No. of circulating lymphoma cells/µL (log10)
10 000
1 000
100
10
1
0.1
0
0.001
0.01
0.1
1
10
100
1 000
B)
From apheresis to day +7
From day +14 to month +3
From month +4 to end of follow up
p < .001
r = -0.3
No. of circulating CAR-T cells/µL (log10)
No. of circulating B cells/µL (log10)
C)
Circulating CAR-T cells
Circulating lymphoma cells
% of patients with circulating lymphoma cells and/or CAR-T cells
100
80
60
40
20
0
Aph. N=58
Pre-Inf. N=56
D +1 N=42
D +3 N=33
D +7 N=58
D +14 N=58
D +21 N=58
M +1 N=58
M +2 N=51
M +3 N=40
M +4 N=33
M +5 N=28
M +6 N=33
M +7 N=25
M +8 N=25
M +9 N=23
M +10 N=24
M +11 N=19
M +12 N=28
M +15 N=21
M +18 N=20
M +21 N=16
Y +2 N=17
Y +2.5 N=1
Y +3 N=7
Y +3.5 N=2
Y +4 N=2
Y +4.5 N=1
Time since CAR-T cell infusion
D)
Circulating CAR-T cells
Circulating normal B lymphocytes
% of patients with normal B lymphocytes and/or CAR-T cells
Time since CAR-T cell infusion
E)
N = 10
No. of circulating B cells/µL (log10)
1 000
100
10
5
1
0.1
0.01
0
No. of circulating CAR-T cells/µL
>25
20
15
10
5
0
+1 +2 +3 +4 +5 +6 +7 +8 +9 +10 +11 +12 +15 +18 +21 +24 +30 +36 +42 +48 +54
Months since CAR-T cell infusion
F)
N = 8
Months since CAR-T cell infusion

Figure S4

Variables	DLBCL Patients: Partial Response/ No Response (N=25)	DLBCL Patients: Complete Response (N=33)	Impact on Complete Response (CR): Univariate analysis Odds Ratio (95%CI)	Univariate analysis *p value*	Multivariate analysis Odds Ratio (95%CI)	Multivariate analysis *p value*
**Demographics**						
**Age**, years	62 (34-76)*	62 (32-79)*				
< 65	16/33 (48%)	17/33 (52%)				
≥ 65	9/25 (36%)	16/25 (64%)	1.7 (0.6-4.9)	*NS*		
**Gender**						
Male	18/36 (50%)	18/36 (50%)				
Female	7/22 (32%)	15/22 (68%)	2.1 (0.7-6.5)	*NS*		
**Pre-apheresis**						
**Previous therapy**						
< 3 lines	16/47 (34%)	31/47 (66%)				
≥ 3 lines	9/11 (82%)	2/11 (18%)	0.1 (0.02-0.6)	*0.01*		
**Previous aHSCT**						
No	22/47 (47%)	25/47 (53%)				
Yes	3/11 (27%)	8/11 (73%)	2.4 (0.6-10)	*NS*		
**Disease status**						
Non-refractory to last line of therapy	2/20 (10%)	18/20 (90%)			11.3 (1.7-74)	*0.01*
Refractory to last line of therapy	23/38 (61%)	15/38 (39%)	0.07 (0.02-0.4)	*<0.001*		
**Circulating lymphoma cells (CLC)**						
No	21/48 (44%)	27/48 (56%)				
Yes	4/8 (50%)	4/8 (50%)	0.8 (0.2-3.5)	*NS*		
**Bulky mass** ≥ 7 cm						
No	18/45 (40%)	27/45 (60%)				
Yes	7/13 (54%)	6/13 (46%)	0.6 (0.2-2)	*NS*		
**IPI**						
< 3 risk factors	15/36 (42%)	21/36 (58%)				
≥ 3 risk factors	10/22 (45%)	12/22 (55%)	0.8 (0.3-2.5)	*NS*		
**ECOG performance status**						
0	4/17 (24%)	13/17 (76%)				
1-2	21/41 (51%)	20/41 (49%)	0.3 (0.08-1.1)	*0.05*		
**Disease stage**						
I / II	7/18 (39%)	11/18 (61%)				
III / IV	18/40 (45%)	22/40 (55%)	0.8 (0.3-2.4)	*NS*		
**LDH,** IU/L	258 (174-1219)*	259 (127-1934)*				
Normal (< 280)	8/23 (35%)	15/23 (65%)				
Elevated (≥ 280)	17/35 (49%)	18/35 (51%)	0.6 (0.2-1.7)	*NS*		
**Ferritin,** ng/mL	627 (106-2165)*	422 (55-1642)*				
Normal (< 300 in male and < 200 in female)	6/18 (37%)	12/18 (67%)				
Elevated (≥ 300 in male and ≥ 200 in female)	19/39 (49%)	20/39 (51%)	0.5 (0.2-1.7)	*NS*		
**Detectable B cells in blood**						
No	22/43 (51%)	21/43 (49%)				
Yes	3/14 (21%)	11/14 (79%)	3.8 (0.9-16)	*0.05*		
**Pre-lymphodepletion**						
**Bulky mass** ≥ 7 cm						
No	17/45 (38%)	28/45 (62%)				
Yes	8/13 (62%)	5/13 (38%)	0.4 (0.1-1.4)	*NS*		
**IPI**						
< 3 risk factors	13/33 (39%)	20/33 (61%)				
≥ 3 risk factors	12/22 (55%)	10/22 (45%)	0.5 (0.2-1.6)	*NS*		
**Disease stage**						
I / II	4/15 (27%)	11/15 (73%)				
III / IV	21/40 (52%)	19/40 (48%)	0.3 (0.09-1.2)	*NS*		
**MTV,** cm^3	149 (3.3-2539)*	46 (0-1699)*				
< 85	7/25 (28%)	18/25 (72%)				
≥ 85	16/29 (55%)	13/29 (45%)	0.3 (0.1-1)	*0.04*		
**TLG,** SUV x cm^3	1094 (20-27214)*	285 (0-11927)*				
< 62	3/17 (18%)	14/17 (82%)				
≥ 62	18/37 (49%)	19/37 (51%)	0.2 (0.05-0.7)	*0.01*		
**Bone marrow involvement**						
Negative	18/40 (45%)	22/40 (55%)				
Positive	7/18 (39%)	11/18 (61%)	1.3 (0.4-4)	*NS*		
**Extranodal involvement**						
Negative	6/25 (24%)	19/25 (76%)				
Positive	19/32 (59%)	13/32 (41%)	0.2 (0.07-0.7)	*0.01*		
**LDH,** IU/L	343 (132-1013)*	224 (134-958)*				
Normal (< 280)	7/28 (25%)	21/28 (75%)				
Elevated (≥ 280)	18/30 (60%)	12/30 (40%)	0.2 (0.07-0.7)	*0.01*		
**Ferritin,** ng/mL	809 (60-2588)*	365 (15-4432)*				
Normal (< 300 in male and < 200 in female)	4/18 (22%)	14/18 (78%)				
Elevated (≥ 300 in male and ≥ 200 in female)	21/40 (53%)	19/40 (47%)	0.3 (0.07-0.9)	*0.03*		
**Pre-infusion**						
**Ferritin,** ng/mL	951 (42-4704)*	535 (14-5217)*				
Normal (< 300 in male and < 200 in female)	5/13 (38%)	8/13 (62%)				
Elevated (≥ 300 in male and ≥ 200 in female)	20/45 (44%)	25/45 (56%)	0.8 (0.2-2.8)	*NS*		
**Circulating lymphoma cells (CLC)**						
No	22/51 (43%)	29/51 (57%)				
Yes	3/6 (50%)	3/6 (50%)	0.8 (0.1-4.1)	*NS*		
**Detectable B cells in blood**						
No	25/52 (48%)	27/52 (52%)				
Yes	0/6 (0%)	6/6 (100%)	0.5 (0.4-0.7)	*0.03*		

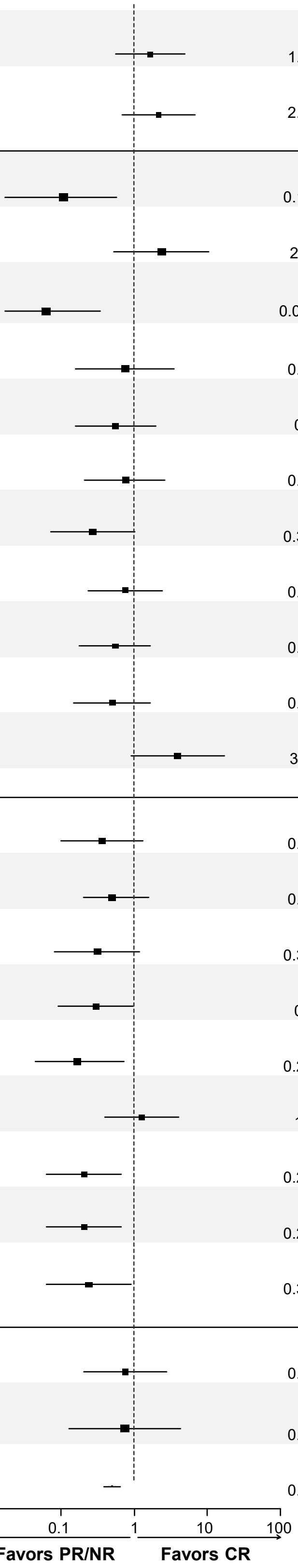

Variables	DLBCL Patients: Partial Response/ No Response (N=25)	DLBCL Patients: Complete Response (N=33)	Impact on Complete Response (CR): Univariate analysis Odds Ratio (95%CI)	Univariate analysis *p value*	Multivariate analysis Odds Ratio (95%CI)	Multivariate analysis *p value*
**CAR-T cell-associated parameters**						
**Product infused (≥ 60 years only)**						
tisa-cel	13/19 (68%)	6/19 (22%)				
axi-cel	2/13 (15%)	11/13 (85%)	12 (2-71)	*0.004*		
**No. of CAR-T cells/μL day +7**	14 (0-134)*	66 (0.06-1771)*				
< 45	20/30 (67%)	10/30 (33%)				
≥ 45	5/27 (19%)	22/27 (81%)	8.8 (2.6-30)	*<0.001*		
**No. of CAR-T cells/μL at peak**	29 (0.5-603)*	132 (5.2-1771)*				
< 60	16/25 (64%)	9/25 (36%)				
≥ 60	9/33 (27%)	24/33 (73%)	4.7 (1.6-15)	*0.01*		
**CAR-Tγδ⁺ cells/μL at CAR-T peak**	<0.001 (<0.001-6)*	0.2 (<0.001-13)*				
Absent (< 0.02)	17/23 (74%)	6/23 (26%)				
Present (≥ 0.02)	8/35 (23%)	27/35 (77%)	9.6 (2.8-32)	*<0.001*		
**AUC days 0 – 28,** days x cells/μL	270 (5-8469)*	1505 (53-12730)*				
< 300	14/18 (78%)	4/18 (22%)				
≥ 300	10/39 (26%)	29/39 (74%)	10 (2.7-38)	*<0.001*		
**CAR-T subpopulations**						
**CAR-Tαβ CD4⁺**, cells/μL	15 (0.1-429)*	46 (<0.001-622)*				
< 10	11/15 (73%)	4/15 (27%)				
≥ 10	14/43 (33%)	29/43 (67%)	5.7 (1.5-21)	*0.01*		
**CAR-Tαβ CD4⁺ CM,** cells/μL	5.3 (0.09-234)*	25 (<0.001-493)*				
< 8.7	16/21 (76%)	5/21 (24%)				
≥ 8.7	9/37 (24%)	28/37 (76%)	9.6 (2.8-35)	*<0.001*		
**CAR-Tαβ CD4⁺ TM,** cells/μL	1.1 (<0.001-186)*	4.1 (<0.001-58)*				
< 1.4	15/23 (65%)	8/23 (35%)				
≥ 1.4	10/35 (29%)	25/35 (71%)	4.7 (1.5-14)	*0.01*		
**CAR-Tαβ CD4⁺ EM,** cells/μL	1 (<0.001-40)*	6.4 (<0.001-115)*				
< 2	14/22 (64%)	8/22 (36%)				
≥ 2	11/36 (31%)	25/36 (69%)	4 (1.3-12)	*0.01*		
**CAR-TFH Th1 like,** cells/μL	<0.001 (<0.001-2.2)*	0.05 (<0.001-6.2)*				
< 0.05	22/39 (56%)	17/39 (44%)				
≥ 0.05	3/19 (16%)	16/19 (84%)	6.9 (1.7-28)	*0.003*		
**CAR-Th1,** cells/μL	4.3 (<0.001-130)*	22 (<0.001-470)*				
< 11	19/29 (66%)	10/29 (34%)				
≥ 11	6/29 (21%)	23/29 (79%)	7.3 (2.2-24)	*<0.001*		
**CAR-Th1 CM,** cells/μL	2.6 (<0.001-89)*	15 (<0.001-366)*				
< 4.2	15/20 (75%)	5/20 (25%)				
≥ 4.2	10/38 (26%)	28/38 (74%)	8.4 (2.4-29)	*<0.001*		
**CAR-Th1 TM,** cells/μL	0.5 (<0.001-64)*	2.9 (<0.001-38)*				
< 1.4	20/30 (67%)	10/30 (33%)				
≥ 1.4	5/28 (18%)	23/28 (82%)	9.2 (2.7-31)	*<0.001*	6.2 (1.4-29)	*0.02*
**CAR-Th1/Th2,** cells/μL	1.8 (<0.001-321)*	9.9 (<0.001-157)*				
< 3	16/25 (64%)	9/25 (36%)				
≥ 3	9/33 (27%)	24/33 (73%)	4.7 (1.6-15)	*0.006*		
**CAR-Th1/Th2 CM,** cells/μL	1.3 (<0.001-198)*	7 (<0.001-149)*				
< 2.6	17/26 (65%)	9/26 (35%)				
≥ 2.6	8/32 (25%)	24/32 (75%)	5.7 (1.8-18)	*0.002*		
**CAR-Th1/Th2 TM,** cells/μL	0.2 (<0.001-122)*	0.6 (<0.001-23)*				
< 0.3	17/29 (59%)	12/29 (41%)				
≥ 0.3	8/29 (28%)	21/29 (72%)	3.7 (1.2-11)	*0.02*		
**CAR-Th1/Th17,** cells/μL	<0.001 (<0.001-14)*	<0.001 (<0.001-4.8)*				
< 0.2	15/26 (58%)	11/26 (42%)				
≥ 0.2	10/32 (31%)	22/32 (69%)	3 (1-8.8)	*0.04*		
**CAR-Th1/Th17 CM,** cells/μL	<0.001 (<0.001-1.2)*	0.1 (<0.001-15)*				
< 0.2	20/38 (53%)	18/38 (47%)				
≥ 0.2	5/20 (25%)	15/20 (75%)	3.3 (1-11)	*0.04*		
**CAR-T CD183⁺CD194⁺CD196⁺CCR10⁻** , cells/μL	<0.001 (<0.001-20)*	0.6 (<0.001-19)*				
< 0.2	16/27 (59%)	11/27 (41%)				
≥ 0.2	9/31 (29%)	22/31 (71%)	3.6 (1.2-11)	*0.02*		
**CAR-T CD183⁺CD194⁺CD196⁺CCR10⁻ CM,** cells/μL	<0.001 (<0.001-5.7)*	0.3 (<0.001-13)*				
< 0.03	16/24 (67%)	8/24 (33%)				
≥ 0.03	9/34 (26%)	25/34 (74%)	5.6 (1.8-17)	*0.003*		
**CAR-Tαβ CD8⁺**, cells/μL	9.2 (<0.001-501)*	53 (0.4-1220)*				
< 13.3	17/23 (74%)	6/23 (26%)				
≥ 13.3	8/35 (23%)	27/35 (77%)	9.6 (2.8-32)	*<0.001*		
**CAR-Tαβ CD8⁺ CM,** cells/μL	4.4 (<0.001-474)*	33 (0.4-997)*				
< 9.1	17/23 (74%)	6/23 (26%)				
≥ 9.1	8/35 (23%)	27/35 (77%)	9.6 (2.8-32)	*<0.001*		
**CAR-Tαβ CD8⁺ TM,** cells/μL	1 (<0.001-292)*	8.3 (0.4-553)*				
< 6	20/33 (61%)	13/33 (39%)				
≥ 6	5/25 (20%)	20/25 (80%)	6.2 (1.9-21)	*0.002*		
**CAR-Tγδ⁺**, cells/μL	<0.001 (<0.001-6)*	0.2 (<0.001-13)*				
< 0.06	21/30 (70%)	9/30 (30%)				
≥ 0.06	4/28 (14%)	24/28 (86%)	14 (3.8-52)	*<0.001*	8.8 (1.9-41)	*0.008*
**CAR-Tγδ⁺ CM,** cells/μL	<0.001 (<0.001-2.1)*	0.2 (<0.001-9.8)*				
< 0.05	21/30 (70%)	9/30 (30%)				
≥ 0.05	4/28 (14%)	24/28 (86%)	14 (3.8-52)	*<0.001*		
**CAR-Tγδ⁺ TM,** cells/μL	<0.001 (<0.001-2.3)*	0.006 (<0.001-3.2)*				
< 0.003	22/38 (58%)	16/38 (42%)				
≥ 0.003	3/20 (15%)	17/20 (85%)	7.8 (2-31)	*0.002*		

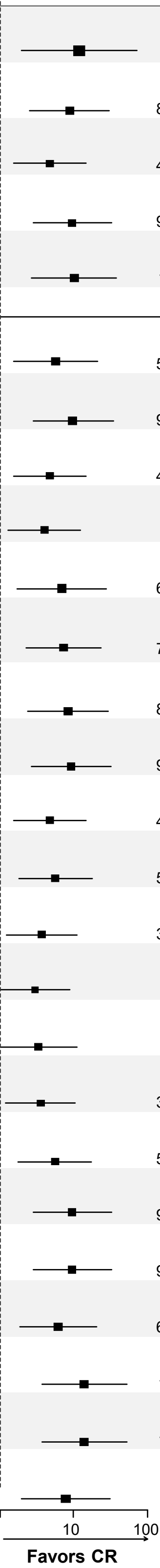

Figure S5

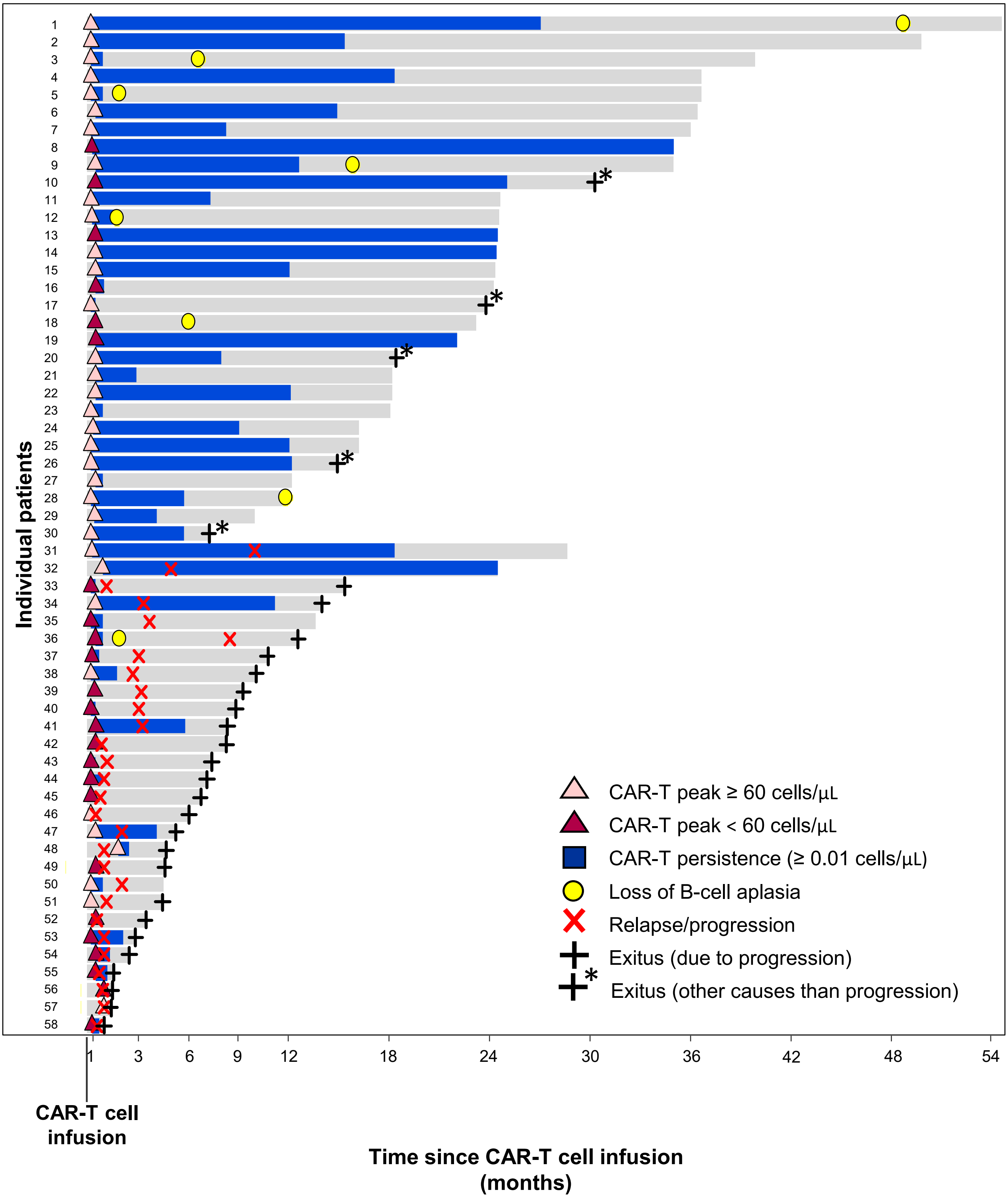
Individual patients
1
2
3
4
5
6
7
8
9
10
11
12
13
14
15
16
17
18
19
20
21
22
23
24
25
26
27
28
29
30
31
32
33
34
35
36
37
38
39
40
41
42
43
44
45
46
47
48
49
50
51
52
53
54
55
56
57
58
CAR-T peak ≥ 60 cells/µL
CAR-T peak < 60 cells/µL
CAR-T persistence (≥ 0.01 cells/µL)
Loss of B-cell aplasia
Relapse/progression
Exitus (due to progression)
Exitus (other causes than progression)
1
3
6
9
12
18
24
30
36
42
48
54
CAR-T cell infusion
Time since CAR-T cell infusion (months)

Figure S6

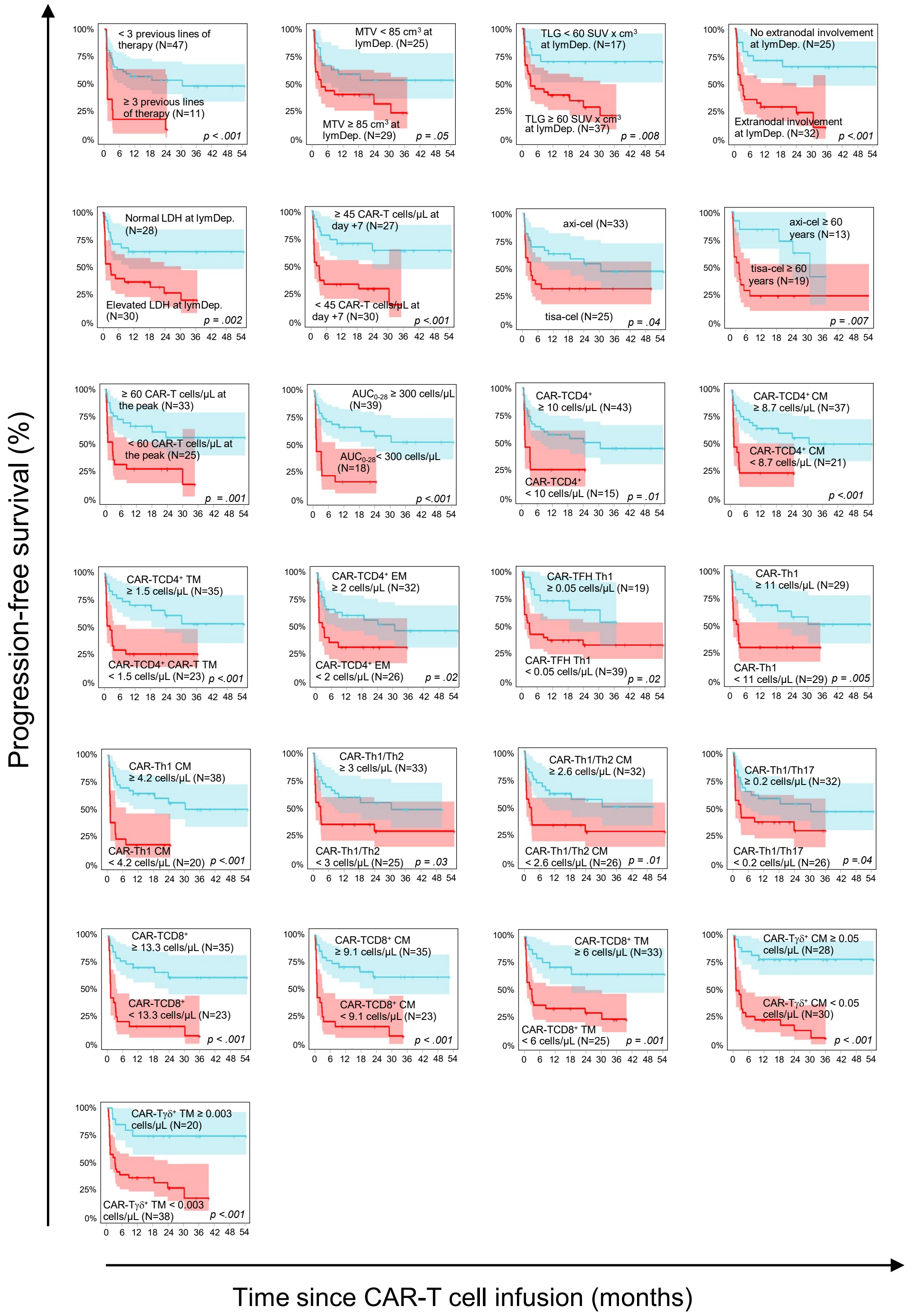
Progression-free survival (%)
Time since CAR-T cell infusion (months)
100%
75%
50%
25%
0%
0 6 12 18 24 30 36 42 48 54
< 3 previous lines of therapy (N=47)
≥ 3 previous lines of therapy (N=11)
p < .001
MTV < 85 cm³ at lymDep. (N=25)
MTV ≥ 85 cm³ at lymDep. (N=29)
p = .05
TLG < 60 SUV x cm³ at lymDep. (N=17)
TLG ≥ 60 SUV x cm³ at lymDep. (N=37)
p = .008
No extranodal involvement at lymDep. (N=25)
Extranodal involvement at lymDep. (N=32)
p <.001
Normal LDH at lymDep. (N=28)
Elevated LDH at lymDep. (N=30)
p = .002
≥ 45 CAR-T cells/µL at day +7 (N=27)
< 45 CAR-T cells/µL at day +7 (N=30)
p <.001
axi-cel (N=33)
tisa-cel (N=25)
p = .04
axi-cel ≥ 60 years (N=13)
tisa-cel ≥ 60 years (N=19)
p = .007
≥ 60 CAR-T cells/µL at the peak (N=33)
< 60 CAR-T cells/µL at the peak (N=25)
p = .001
AUC0-28 ≥ 300 cells/µL (N=39)
AUC0-28 < 300 cells/µL (N=18)
p <.001
CAR-TCD4+ ≥ 10 cells/µL (N=43)
CAR-TCD4+ < 10 cells/µL (N=15)
p = .01
CAR-TCD4+ CM ≥ 8.7 cells/µL (N=37)
CAR-TCD4+ CM < 8.7 cells/µL (N=21)
p <.001
CAR-TCD4+ TM ≥ 1.5 cells/µL (N=35)
CAR-TCD4+ CAR-T TM < 1.5 cells/µL (N=23)
p <.001
CAR-TCD4+ EM ≥ 2 cells/µL (N=32)
CAR-TCD4+ EM < 2 cells/µL (N=26)
p = .02
CAR-TFH Th1 ≥ 0.05 cells/µL (N=19)
CAR-TFH Th1 < 0.05 cells/µL (N=39)
p = .02
CAR-Th1 ≥ 11 cells/µL (N=29)
CAR-Th1 < 11 cells/µL (N=29)
p = .005
CAR-Th1 CM ≥ 4.2 cells/µL (N=38)
CAR-Th1 CM < 4.2 cells/µL (N=20)
p <.001
CAR-Th1/Th2 ≥ 3 cells/µL (N=33)
CAR-Th1/Th2 < 3 cells/µL (N=25)
p = .03
CAR-Th1/Th2 CM ≥ 2.6 cells/µL (N=32)
CAR-Th1/Th2 CM < 2.6 cells/µL (N=26)
p = .01
CAR-Th1/Th17 ≥ 0.2 cells/µL (N=32)
CAR-Th1/Th17 < 0.2 cells/µL (N=26)
p =.04
CAR-TCD8+ ≥ 13.3 cells/µL (N=35)
CAR-TCD8+ < 13.3 cells/µL (N=23)
p < .001
CAR-TCD8+ CM ≥ 9.1 cells/µL (N=35)
CAR-TCD8+ CM < 9.1 cells/µL (N=23)
p < .001
CAR-TCD8+ TM ≥ 6 cells/µL (N=33)
CAR-TCD8+ TM < 6 cells/µL (N=25)
p = .001
CAR-Tγδ+ CM ≥ 0.05 cells/µL (N=28)
CAR-Tγδ+ CM < 0.05 cells/µL (N=30)
p < .001
CAR-Tγδ+ TM ≥ 0.003 cells/µL (N=20)
CAR-Tγδ+ TM < 0.003 cells/µL (N=38)
p <.001

Figure S7

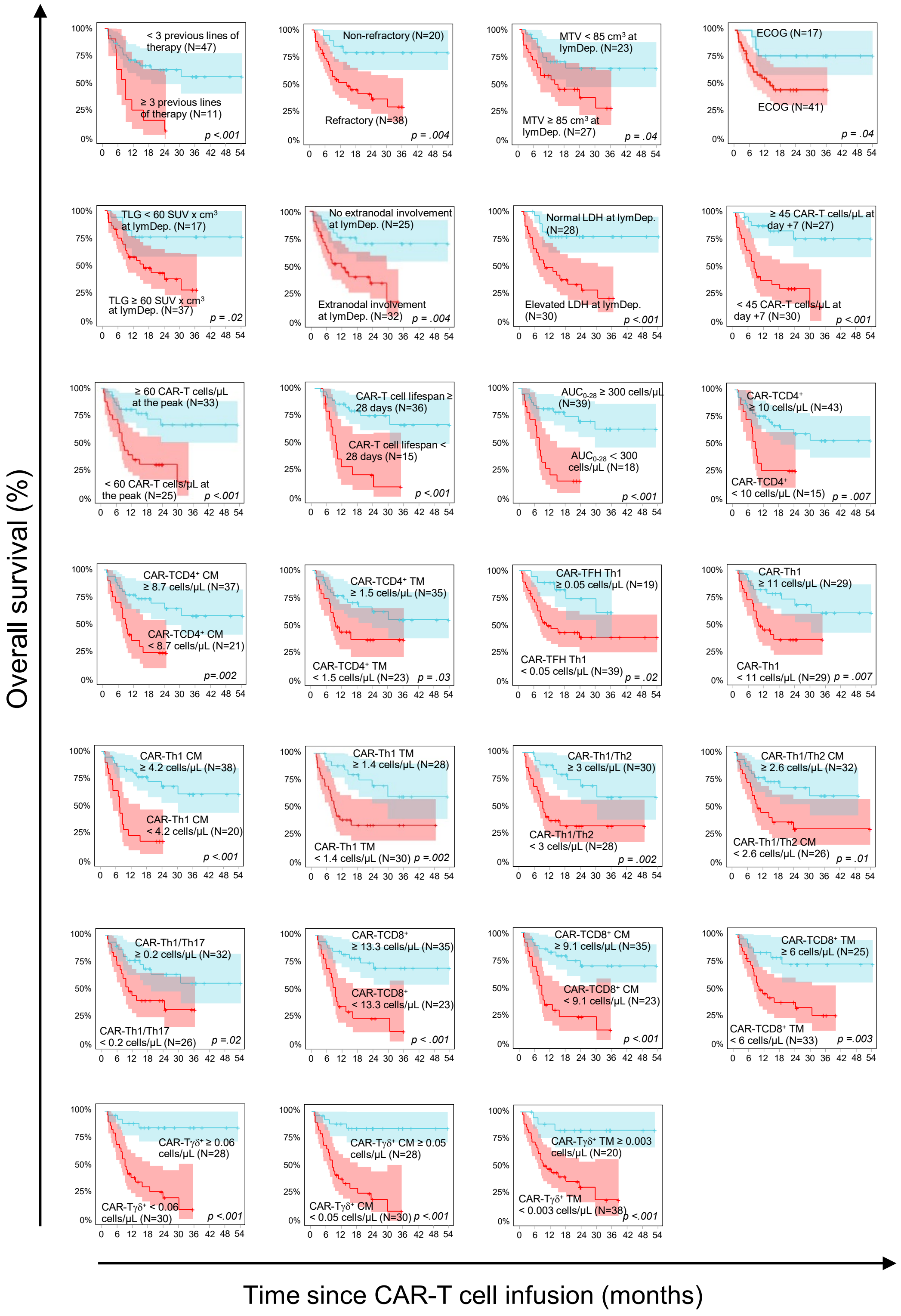
Overall survival (%)
Time since CAR-T cell infusion (months)
< 3 previous lines of therapy (N=47)
≥ 3 previous lines of therapy (N=11)
p <.001
Non-refractory (N=20)
Refractory (N=38)
p = .004
MTV < 85 cm³ at lymDep. (N=23)
MTV ≥ 85 cm³ at lymDep. (N=27)
p = .04
ECOG (N=17)
ECOG (N=41)
p = .04
TLG < 60 SUV x cm³ at lymDep. (N=17)
TLG ≥ 60 SUV x cm³ at lymDep. (N=37)
p = .02
No extranodal involvement at lymDep. (N=25)
Extranodal involvement at lymDep. (N=32)
p = .004
Normal LDH at lymDep. (N=28)
Elevated LDH at lymDep. (N=30)
p <.001
≥ 45 CAR-T cells/μL at day +7 (N=27)
< 45 CAR-T cells/μL at day +7 (N=30)
p <.001
≥ 60 CAR-T cells/μL at the peak (N=33)
< 60 CAR-T cells/μL at the peak (N=25)
p <.001
CAR-T cell lifespan ≥ 28 days (N=36)
CAR-T cell lifespan < 28 days (N=15)
p <.001
AUC0-28 ≥ 300 cells/μL (N=39)
AUC0-28 < 300 cells/μL (N=18)
p <.001
CAR-TCD4+ ≥ 10 cells/μL (N=43)
CAR-TCD4+ < 10 cells/μL (N=15)
p = .007
CAR-TCD4+ CM ≥ 8.7 cells/μL (N=37)
CAR-TCD4+ CM < 8.7 cells/μL (N=21)
p=.002
CAR-TCD4+ TM ≥ 1.5 cells/μL (N=35)
CAR-TCD4+ TM < 1.5 cells/μL (N=23)
p = .03
CAR-TFH Th1 ≥ 0.05 cells/μL (N=19)
CAR-TFH Th1 < 0.05 cells/μL (N=39)
p = .02
CAR-Th1 ≥ 11 cells/μL (N=29)
CAR-Th1 < 11 cells/μL (N=29)
p = .007
CAR-Th1 CM ≥ 4.2 cells/μL (N=38)
CAR-Th1 CM < 4.2 cells/μL (N=20)
p <.001
CAR-Th1 TM ≥ 1.4 cells/μL (N=28)
CAR-Th1 TM < 1.4 cells/μL (N=30)
p =.002
CAR-Th1/Th2 ≥ 3 cells/μL (N=30)
CAR-Th1/Th2 < 3 cells/μL (N=28)
p = .002
CAR-Th1/Th2 CM ≥ 2.6 cells/μL (N=32)
CAR-Th1/Th2 CM < 2.6 cells/μL (N=26)
p = .01
CAR-Th1/Th17 ≥ 0.2 cells/μL (N=32)
CAR-Th1/Th17 < 0.2 cells/μL (N=26)
p =.02
CAR-TCD8+ ≥ 13.3 cells/μL (N=35)
CAR-TCD8+ < 13.3 cells/μL (N=23)
p < .001
CAR-TCD8+ CM ≥ 9.1 cells/μL (N=35)
CAR-TCD8+ CM < 9.1 cells/μL (N=23)
p <.001
CAR-TCD8+ TM ≥ 6 cells/μL (N=25)
CAR-TCD8+ TM < 6 cells/μL (N=33)
p =.003
CAR-Tγδ+ ≥ 0.06 cells/μL (N=28)
CAR-Tγδ+ < 0.06 cells/μL (N=30)
p <.001
CAR-Tγδ+ CM ≥ 0.05 cells/μL (N=28)
CAR-Tγδ+ CM < 0.05 cells/μL (N=30)
p <.001
CAR-Tγδ+ TM ≥ 0.003 cells/μL (N=20)
CAR-Tγδ+ TM < 0.003 cells/μL (N=38)
p <.001
